# G-protein-coupled receptor 141 mediates breast cancer proliferation and metastasis by regulating oncogenic mediators and the p-mTOR/p53 axis

**DOI:** 10.18632/oncotarget.28433

**Published:** 2023-05-19

**Authors:** Monalisa Parija, Amit K. Adhya, Sandip K. Mishra

**Affiliations:** ^1^Cancer Biology Lab, Gene Function and Regulation Group, Institute of Life Sciences, Chandrasekharpur, Bhubaneswar 751023, Odisha, India; ^2^Regional Centre for Biotechnology, NCR Biotech Science Cluster, Faridabad 121001, Haryana (NCR Delhi), India; ^3^Department of Pathology, All India Institute of Medical Sciences, Bhubaneswar 751019, Odisha, India

**Keywords:** G-protein-coupled receptor 141, cell migration, metastasis, p-mTOR, p53

## Abstract

Breast cancer morbidity is surging towards the peak in females across the globe. An inherent property of cancer cells is enhanced cell proliferation rate and migration capability, leading to deregulated cell signaling cascades. G-protein-coupled receptors (GPCRs) have recently emerged as a hot-spot target in cancer research. We identify aberrant expression of G-protein-coupled receptor 141 (GPR141) in different breast cancer subtypes that correlate with poor prognosis. However, the molecular mechanism via which GPR141 advances breast cancer remains elusive. Increased GPR141 expression enhances the migratory behavior of breast cancer, driving oncogenic pathways both *in vitro* and *in vivo* through activation of epithelial to mesenchymal transition (EMT), oncogenic mediators and regulation of p-mTOR/p53 signaling. Our study unveils a molecular mechanism for p53 downregulation and activation of p-mTOR1 and its substrates in GPR141 overexpressed cells, accelerating breast tumorigenesis. We find that an E3 ubiquitin ligase, Cullin1, partly mediates p53 degradation via proteasomal pathway. Co-immunoprecipitation result shows that the phosphorylated form of 40S ribosome protein S6 (ps6., a p-mTOR1 substrate) forms a complex with Cullin1. These findings suggest an interplay between Cullin1 and p-mTOR1 in GPR141 overexpressed cells that downregulates p53 expression, thus inducing tumor growth. GPR141 silencing restores p53 expression and attenuates p-mTOR1 signaling events, thereby impeding proliferation and migration in breast cancer cells. Our findings describe the role of GPR141 in breast cancer proliferation, and metastasis, as well as in influencing the tumor microenvironment. Modulating GPR141 expression could pave the way for a better therapeutic approach to regulating breast cancer progression and metastasis.

## INTRODUCTION

Breast cancer contributes to the highest number of cancer deaths in women globally than any other malady. Breast cancer incidence will rise from 203.5 per 100 000 females in 2011 to 233 per 1000 females in 2026 [1]. Cancer cells undergo uncontrolled cellular proliferation that triggers tumor development. The transformation of healthy to tumor cells is stimulated by multiple factors such as alteration in the tumor microenvironment, change in morphology, and enhanced proliferative, migratory capacity. Metastasis represents a multimodal cascade of signaling events that involve the dissemination of cancer cells from *in situ* neoplasia to the secondary sites. More than 90 percent of cancer-related mortality is frequently attributed to metastasis [2]. Epithelial to mesenchymal (EMT) status has been positively correlated to the behavior of metastatic lesions and overall survival [3].

The tumor niche contains a variety of cell types that express GPCRs namely, stromal, circulatory, and immunological cells [4]. GPCRs are the most prominent family of cell surface receptors in humans [4]. Approximately 4% of the genetic code of human are expressed by over a thousand GPCRs [5]. GPCRs govern multiple tumorigenesis processes, including cell proliferation, metastasis, angiogenesis, and chemoresistance [5]. GPCRs have evolved as the principal candidates for successful curatives contributing to 50% of pharmaceutical targets, owing to their broad expressivity on cellular surfaces and diverse physiological functions [6]. GPR141 is a class A orphan receptor molecule of the rhodopsin family [7, 8]. Human fibrosarcoma HT1080 cells and bone marrow both express GPR141 [9]. GPR141 is notably amplified in inflammatory breast cancer [10]. However, its molecular mechanism of driving tumorigenesis is yet to be fully explored.

p53 malfunction and mTOR pathway hyperactivation are attributes of cancer progression [11]. Various reports suggest crosstalk of p53 and the mTOR pathway will provide new insight into the molecular coordination of growth signals and stress response in impeding cancer progression [11]. We illustrated through our data that GPR141 stimulates proliferation by increased expression of mammalian target of rapamycin complex (p-mTOR1) and downregulation of p53. There is a coordinated interplay between cancer cells and tumor microenvironment components [12]. GPR141 also drives tumorigenesis by E-cadherin degradation and MMP7 activation. We tried to delineate how GPR141 modulates breast cancer progression by regulating the p-mTOR/p53 axis. This research uncovers GPR141 as a stimulator of breast tumorigenesis and metastasis, making it a candidate target for breast cancer therapeutics.

## RESULTS

### Enhanced GPR141 expression in breast carcinoma augmenting metastasis and the tumor niche

**Figure 1:**
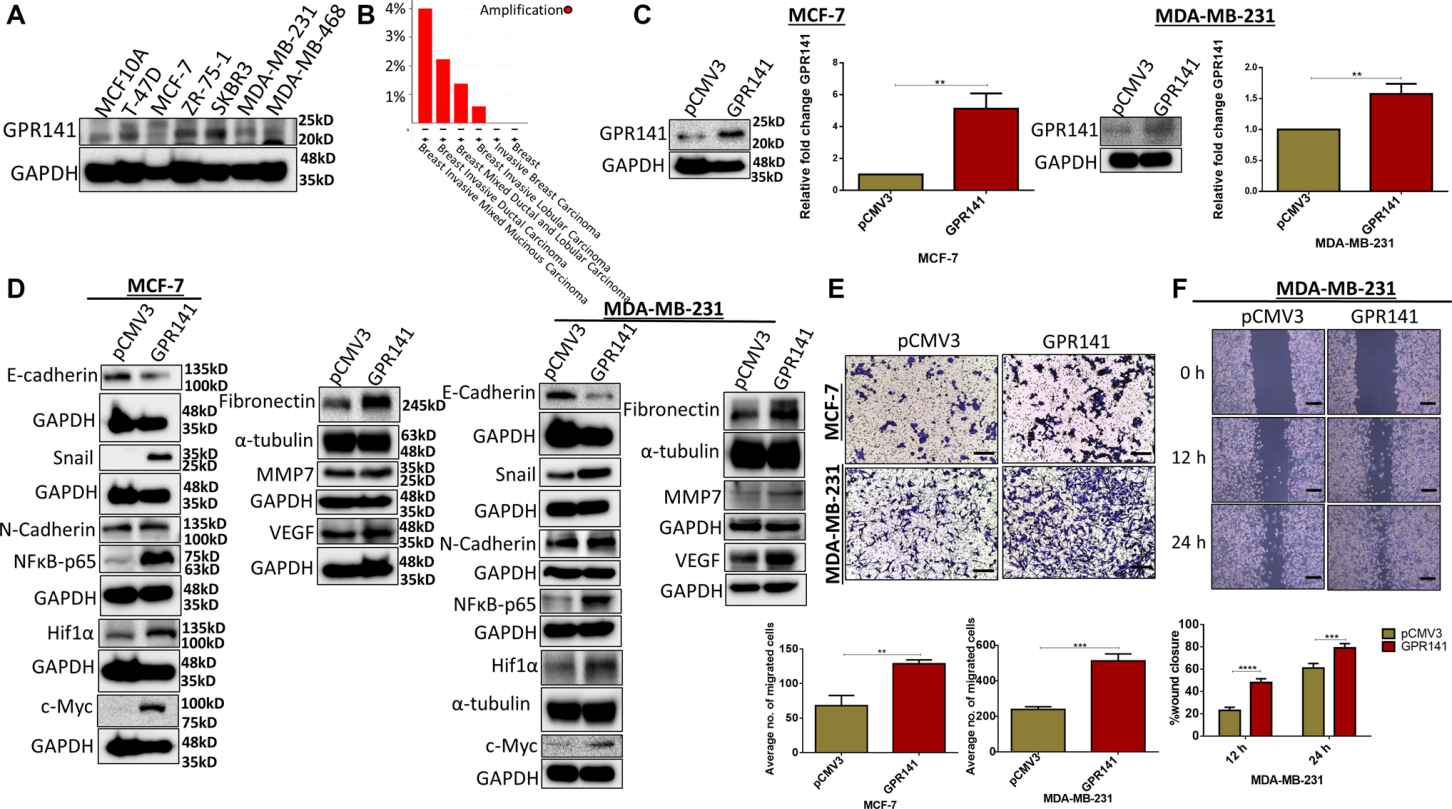
GPR141 overexpression in breast carcinoma induces the expression level of oncogenic mediators and EMT markers and influences tumor niche *in vitro*. (**A**) Immunoblot data show the expression level of GPR141 in different breast cancer cells and healthy breast epithelial MCF10A cells. (**B**) The graph depicts the amplification of GPR141 in different breast cancer subtypes. (**C**) Immunoblot and the relative mRNA expression show the level of GPR141 in ctrl pCMV3 and GPR141 overexpressed MCF-7 and MDA-MB-231 cells. (**D**) Immunoblot exhibiting the expression level of EMT markers and oncogenes. (**E**) Migration assay showing the number of migrated cells through transwell chamber 48 h after stimulation with 5% FBS. Scale bar: 50 μm. Student’s *t*-test was used for the statistical analysis. (**F**) Wound-healing assay showing 0, 12, and 24 h post-scratch and its graphical representation showing percentage wound closure in ctrl pCMV3 and GPR141 overexpressed MDA-MB-231 cells. Scale bar used in this study: 50 μm. Two-way ANOVA was used for the statistical analysis. Overall data are represented as mean ± SD. Student’s *t*-test and two-way ANOVA were used for the statistical analysis, *n* = 3 (^****^ *P* ≤ 0.0001, ^***^ *P* ≤ 0.001, ^**^ *P* ≤ 0.01, Significant).

To determine the status of GPR141 in breast carcinoma, we assessed the expression levels of GPR141 in different breast cancer and healthy breast cell lines with western blot data (Figure 1A) and measured transcript levels of GPR141 (Supplementary Figure 1A, 1B) in these cells. GPR141 amplification was observed in different breast cancer sub-types, with high alteration frequency observed in breast invasive mixed mucinous carcinoma (4% altered out of 25 cases) and breast invasive ductal carcinoma (2.23% in 1660 cases) (Figure 1B). To ascertain GPR141’s potential contribution to *in vitro* breast carcinogenesis, we overexpressed GPR141 in Estrogen receptor positive (MCF-7) and triple-negative breast cancer cells (MDA-MB-231). Immunoblot and transcript data indicate overexpression of GPR141 in both cells (Figure 1C).

The potential of breast cancer metastasizing is continuously escalating. Distant metastasis can result in a steep decline in the 5-year overall survival rate to about 25%, as opposed to 80% for breast cancer patients who do not develop metastasis [13]. Epithelial to mesenchymal transition (EMT) is a trait of cancer. EMT stimulates enhanced tumor development and metastatic property [14]. We checked the role of GPR141 in EMT and its downstream signaling pathways. Immunoblot and Q-RT PCR results showed that GPR141 overexpression stimulates migration through the accumulation of mesenchymal markers such as N-cadherin, Snail, and reduction in the expression of epithelial marker (E-cadherin) in both MCF-7 and MDA-MB-231 cells (Figure 1D and Supplementary Figure 1C). The proangiogenic and antiangiogenic factors work in tandem to control the development of new blood capillaries inducing angiogenesis [15]. Matrix metalloprotease 7 (MMP7) cleaves E-cadherin in normal epithelial cells resulting in diminished adhesion and accelerated proliferation [16]. An increase in the expression of Vascular Endothelial Growth Factor (VEGF) and MMP7 was observed in response to GPR141 overexpression in both MCF-7 and MDA-MB-231 cells (Figure 1D). Furthermore, GPR141 promotes oncogene mediators like fibronectin, Hypoxia Inducible Factor 1α (HIF1α), and c-Myc expression in both MCF-7 and MDA-MB-231 cells (Figure 1D).

To study the involvement of GPR141 in migration, we performed transwell migration (Figure 1E) and wound healing assay (Figure 1F and Supplementary Figure 1D). Results showed increased migration in MCF-7 and MDA-MB-231 cells. The evidence from *in vitro* research indicates that GPR141 exerts a substantial role in breast tumorigenesis.

### GPR141 regulates proliferation in breast cancer cells by modulating p53

**Figure 2:**
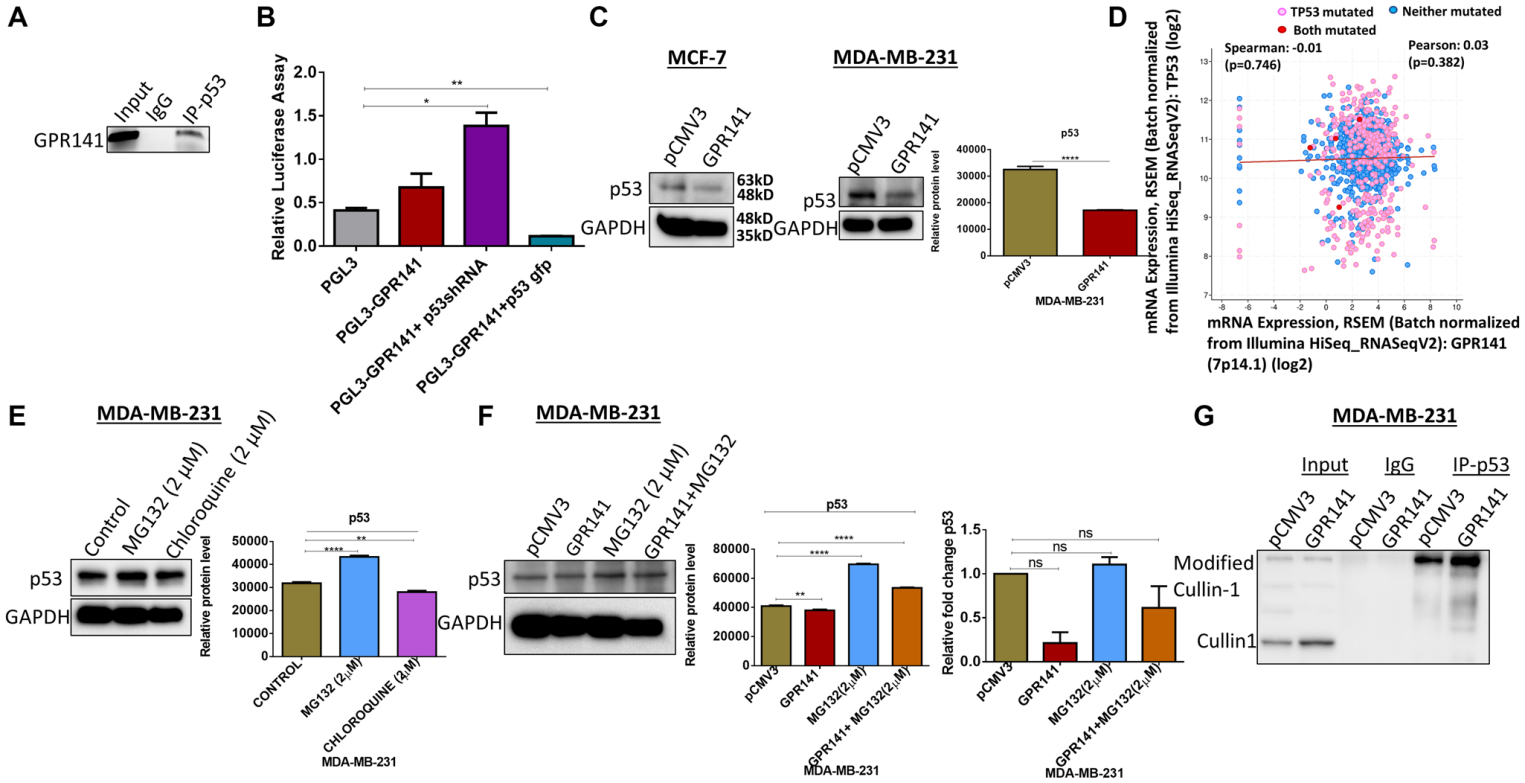
p53 is essential for the proliferative behavior of GPR141 overexpressed cells. (**A**) Chromatin Immunoprecipitation (chIP) data showing the expression of the binding of p53 onto the promoter region of GPR141. (**B**) Luciferase assay revealing differential regulation of promoter activity of GPR141 by p53. (**C**) The expression level of p53 in MCF-7 and MDA-MB-231 cells was analyzed by immunoblot after overexpressing GPR141. Immunoblots are representative of three independent experiments. Densitometry quantification was provided. (**D**) The correlation of TP53 mRNA expressions with GPR141 mRNA expressions in 1084 TCGA breast invasive carcinomas from cBioPortal. (**E**) MDA-MB-231 cells were treated with 2 μM MG132 and Chloroquine independently for 12 h, and the p53 protein expression was analyzed by immunoblotting. A representative immunoblot is shown. GAPDH was used as a loading control. Densitometry quantification with statistical analysis has been provided. (**F**) Representative immunoblot analysis of the p53 in pCMV3, GPR141 overexpressed independently and with MG132. GAPDH was used as a loading control. Relative p53 mRNA expression levels were analyzed using q RT-PCR. Three technical repeats (*N* = 3) were performed, and the data represent the means ± SD. (two-tailed *t*-test; Significant, ns: no significant difference). (**G**) Using immunoblotting, co-immunoprecipitation was performed with an antibody against p53 and analyzed with Cullin1 antibody in both control pCMV3 and GPR141 overexpressed cells. An anti-IgG antibody was used as a negative control. The data are represented as mean ± SD. Student’s *t*-test was used for the statistical analysis, *n* = 3 (^****^ *P* ≤ 0.0001, ^**^ *P* ≤ 0.01, ^*^ *P* ≤ 0.05, Significant). Abbreviation: ns: no significant difference.

Functional genomic analyses of GPR141 reveal that p53 is located close to the locus of chromosome 7, which raises the probability that their expression could be modulated by binding to GPR141. In order to elucidate this, Chromatin Immunoprecipitation (chIP) assay was carried out to demonstrate the interaction of p53 with the GPR141 promoter. p53 is getting recruited to the promoter site of GPR141 (Figure 2A). Moreover, the luciferase assay shows differential regulation of p53 on the GPR141 promoter (Figure 2B). Then, we checked the expression pattern of p53 upon GPR141 overexpression at protein and transcript levels, where p53 downregulation is evident at the protein level in MCF-7 and MDA-MB-231 cells (Figure 2C). No significant differences were observed at the transcript level (Supplementary Figure 2A). Mutations of p53 are attributed to cancer progression. TCGA datasets of 1084 invasive breast carcinoma patient samples from cBioPortal illustrate mRNA expression of TP53 is negatively correlated with GPR141 mRNA expression level (Spearman’s correlation = −0.00987, *P* = 0.746) (Figure 2D). Next, we tried to explore the molecular mechanism behind p53 downregulation. Since proteasome-mediated degradation and lysosome-mediated degradation account for most of the protein degradation processes in eukaryotes, p53 might get regulated by this process. To corroborate the hypothesis, 2 μM of lysosomal inhibitor, chloroquine, and a proteasome inhibitor, MG132, were administered in MDA-MB-231 cells for 12 h to determine the expression of p53 by immunoblotting. Results indicate upregulated p53 expression by MG132 treatment. Densitometry quantification with statistical analysis has been mentioned (Figure 2E). Ubiquitination regulating protein stability and lysis is gaining interest in the realm of medical oncology [17]. MG132 is an inhibitor that prevents 26S proteasome from degrading proteins [18]. Then we treated control pCMV3 and GPR141 overexpressed cells with MG132 and found upregulation of p53 expression in MG132 treated cells. (Figure 2F and Supplementary Figure 2B). Densitometry quantification was provided. No significant differences were observed at the transcript level (Figure 2F and Supplementary Figure 2C). These results suggest that p53 downregulation is partly mediated through the proteasomal degradation pathway.

Cullin-RING ligases (CRLs) contribute approximately 20 percent of total proteolysis in the ubiquitin-proteasome circuit [19]. Cullin1 is a constituent of the E3 ubiquitin ligases family. Cullin1 promotes the ubiquitination of specific proteins, which govern multiple biological and cellular processes, including the cell cycle [20]. Co-immunoprecipitation (Co-IP) data revealed the interaction of p53 with Cullin1 in both control and GPR141 overexpressed MDA-MB-231 and MCF-7 cells (Figure 2G and Supplementary Figure 2D). To summarize, p53 is a significant regulator of cell proliferation, and downregulation of p53 expression mediates tumorigenesis. GPR141 increases p53 degradation partly by a proteasomal pathway mediated via Cullin1.

### GPR141 induces cellular proliferation, cell cycle advancement, and colony formation *in vitro* in breast cancer

**Figure 3:**
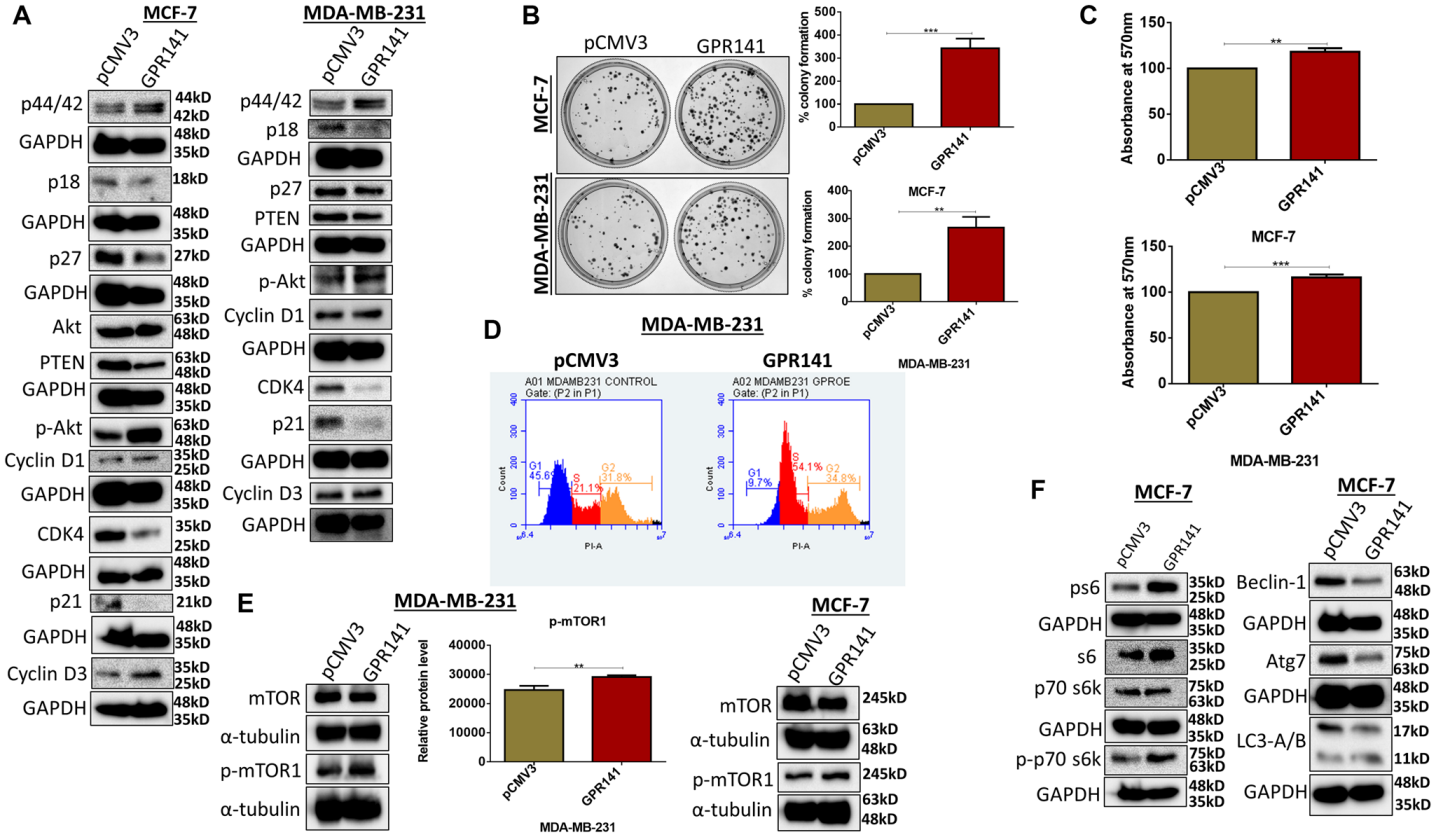
GPR141 stimulates breast cancer proliferation and tumorigenesis *in vitro*. (**A**) Immunoblots showing the expression level of cellular proliferative markers. (**B**) Colony formation assay showing colony numbers per well along with graphical representation. (**C**) Cell proliferation quantified by 3-(4,5-dimethylthiazol-2-yl)-2,5-diphenyl-2H-tetrazolium bromide (MTT) assay after 48 h. (**D**) Fluorescence-activated cell sorting assay (FACS) shows an increase in the G1-S transition phase upon overexpression of GPR141 in MDA-MB-231 cells. (**E**, **F**) Immunoblot showing the phosphorylation levels of p-mTOR1 in MCF-7 and MDA-MB-231 cells after GPR141 overexpression, and its substrates ps6, p-p70 s6kinase T389 followed by autophagy markers expression in MCF-7 cells. The data are represented as mean ± SD. Student’s *t*-test was used for the statistical analysis, *n* = 3 (^***^ *P* ≤ 0.001, ^**^ *P* ≤ 0.01).

Cancer cells circumvent various cell cycle checkpoints leading to enhanced proliferative capability [21]. To comprehend the role of GPR141 in cell cycle regulation, we checked the expression of proliferative markers (CyclinD1, CyclinD3) (Figure 3A), and our results exhibit pronounced cellular proliferation in both MCF-7 and MDA-MB-231 cells. In addition, GPR141 overexpression downregulates CDK4, and p53 downstream targets p27 and p21 (Figure 3A). The proliferative capacity of the overexpressed GPR141 in MCF-7 and MDA-MB-231 cells was assessed through clonogenic assay, cell viability assay, and cell cycle analysis. Results show more colonies upon GPR141 overexpression compared to control cells (Figure 3B), and an increased proliferative rate (Figure 3C). Cell cycle analysis through FACS showed enhanced G1 to S transition compared to control breast cancer cells with the S phase acquiring 54.1% of the cell cycle compared to 21.1% in control MDA-MB-231 cells (Figure 3D and Supplementary Figure 3A).

We checked the phosphorylation levels of kinases in both MCF-7 and MDA-MB-231 cells to elucidate various signaling pathways that mediate GPR141-dependent breast tumorigenesis. GPR141 overexpression enhanced the phosphorylation levels of mTOR1 in MCF-7 and MDA-MB-231 cells shown in immunoblot and densitometric quantification (Figure 3E). cBioPortal downloaded from TCGA datasets of 1084 invasive breast carcinoma patient samples estimated a positive correlation of mRNA expression of mTOR with GPR141 mRNA expression level (Spearman’s correlation = 0.210, *P* = 2.70e-12) (Supplementary Figure 3B). mTOR signaling cascade is constitutively active in tumors to aid in tumor cell proliferation, growth, and metabolic activity [22]. mTOR1 regulates protein translation by phosphorylating its downstream effectors, such as Akt and ps6 [23]. Enhanced phosphorylation of p-p70s6 kinase Thr-389, ps6 (Ser 235/236) (Figure 3F and Supplementary Figure 3C), observed by the downregulation of its negative regulators, such as PTEN (Figure 3A), results in increased protein translation. PTEN is one of the downstream regulators of p53, deregulated upon GPR141 overexpression. Reports suggest that PTEN mutation contributes to tumor development, followed by constitutive activation of the p-mTOR pathway [23]. Autophagy is a lysosomal-dependent cell degradation phenomenon that enables cell survival. mTOR1 negatively regulates autophagy by suppressing beclin1 expression and other autophagy markers (Figure 3F and Supplementary Figure 3C) [24].

### GPR141 promotes breast cancer cells tumorigenesis via the p-mTOR/p53 pathway

**Figure 4:**
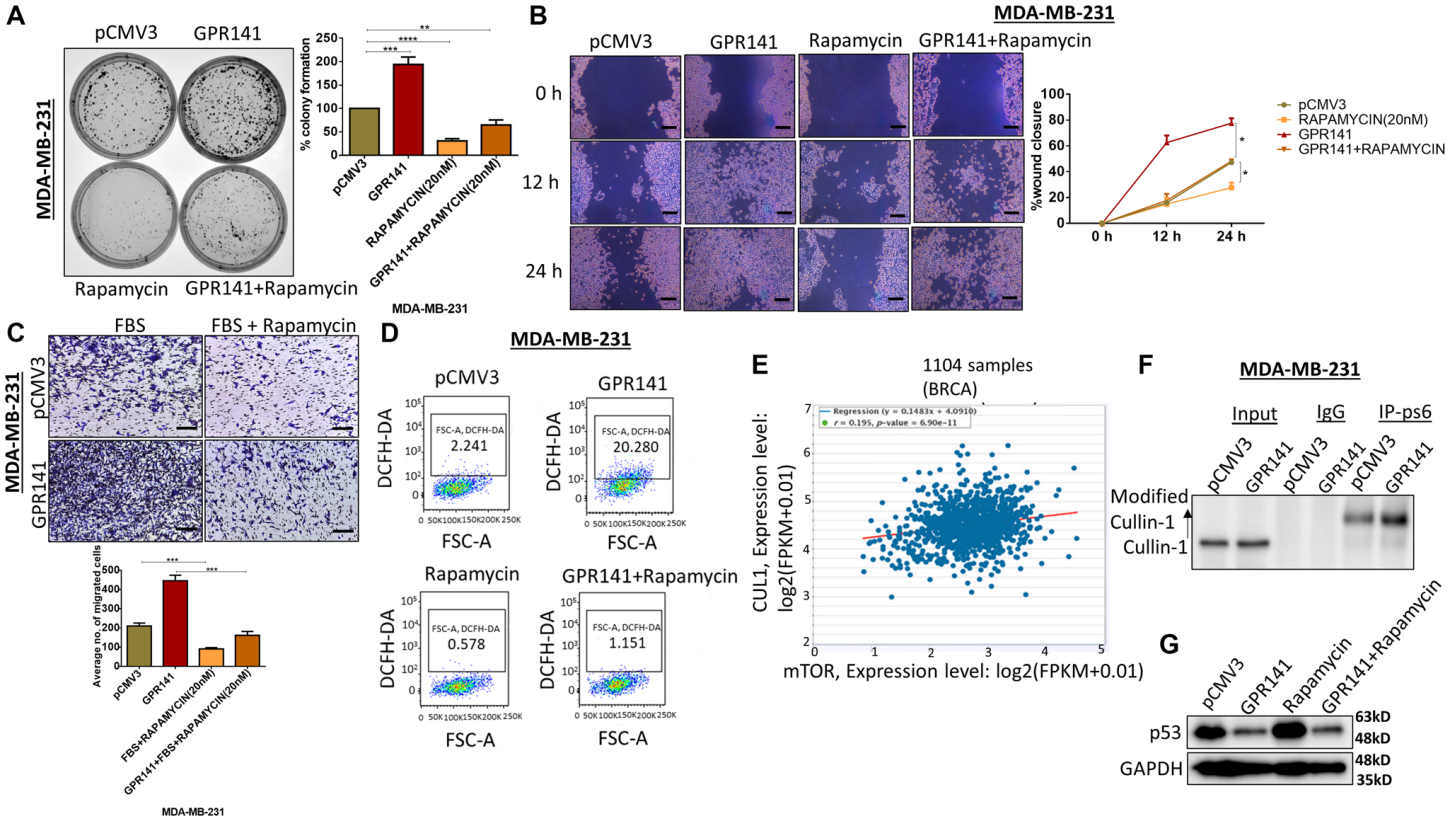
GPR141 overexpression induces breast cancer cell tumorigenesis via the p-mTOR1-p53 pathway. (**A**) Colony forming assay showing the number of colonies developed with or without p-mTOR1 inhibitor, rapamycin. (**B**) Wound-healing/scratch assay showing cell migration after 48 h post rapamycin treatment in control pCMV3 and GPR141 overexpressed cells. Scale bar: 50 μm. (**C**) Migration assay showing the number of invading cells through transwell chamber 48 h after stimulation with 5% FBS in ctrl pCMV3 and GPR141 overexpressed MDA-MB-231 cells with and without rapamycin. Scale bar: 50 μm. (**D**) Reactive Oxygen Species (ROS) level in MDA-MB-231 cells measured through 2′,7′-Dichlorofluorescein diacetate (DCFH-DA) at 24 h after rapamycin treatment. (**E**) The correlation of mTOR mRNA expressions with Cullin1 mRNA expressions in 1104 TCGA breast invasive carcinomas from starBase v3.0 project. (**F**) Coimmunoprecipitation was performed with an antibody against ps6 (p-mTOR1 substrate) and analyzed with Cullin1 antibody in control pCMV3 and overexpressed GPR141 in MDA-MB-231 cells. An anti-IgG antibody was used as a negative control. (**G**) Immunoblot showing the expression level of p53 in control pCMV3 and GPR141 overexpressed cells post rapamycin treatment in both the cells. The data are represented as mean ± SD. Student’s *t*-test was used for the statistical analysis, *n* = 3 (^****^ *P* ≤ 0.0001, ^***^ *P* ≤ 0.001, ^**^ *P* ≤ 0.01, ^*^ *P* ≤ 0.05, Significant).

GPR141 overexpression increased the activation of p-mTOR1 and reduced p53 expression levels in breast cancer cell lines. So, we evaluated the involvement of GPR141 in p-mTOR1/p53 axis signaling in breast tumorigenesis. To identify whether GPR141 mediates the *in vitro* tumorigenesis through the p-mTOR pathway in MCF-7 and MDA-MB-231 cells, we treated the control and GPR141 overexpressed cells with rapamycin. Rapamycin has been reported to limit the growth of cancer cells by suppressing mTOR [25]. We investigated breast cancer cell migration, proliferation, and invasion following treatment with rapamycin. Our results showed a lesser number of colonies formation (Figure 4A and Supplementary Figure 4A), a reduction in cellular migration at 12 h and 24 h post-scratch (Figure 4B), 48 h in transwell migration assay (Figure 4C and Supplementary Figure 4B), proliferation (Supplementary Figure 4C), in MCF-7, MDA-MB-231 cells treated with the inhibitor as compared to control cells suggesting that these functions are mostly driven by p-mTOR1-dependent stimulation in breast carcinoma. Uncontrolled ROS generation drives cancer progression through multiple signaling pathways such as mTOR, PTEN, and MMPs [26]. We checked the ROS level in control, GPR141 overexpressed, and rapamycin-treated cells and the results show that the rapamycin-treated cells exhibit reduction in the ROS level quantified by DCFH-DA staining compared to the higher levels of ROS in GPR141 overexpressed MDA-MB-231 and MCF-7 cells (Figure 4D and Supplementary Figure 4D). According to our findings, the rapamycin-treated group of cells displayed lower levels of cellular viability, proliferation, and migration at different time frames than control breast cancer cells.

Elevated mTORC1 levels in the cells showed high levels of active proteasomes through a broad uptick in the gene expression encoding proteasome subunits [27]. We used the starBase v3.0 project, which contained the gene expression profiles of 1104 TCGA invasive breast carcinomas, to determine the association between CUL1 with mTOR, and revealed a strong positive correlation between CUL1 and mTOR expression levels (*r* = 0.195, *P* = 6.90e-11) (Figure 4E). Simultaneously, we determined the possible interaction between ps6 (p-mTOR1 substrate) and Cullin1, E3 ubiquitin ligase to test this hypothesis. Co-immunoprecipitation data identified the interaction of Cullin1 with ps6 (Figure 4F). We further verified the role of p-mTOR1 signaling in GPR141 mediated tumorigenesis by p53 alteration using the p-mTOR1 inhibitor, rapamycin, and found restoration of p53 in GPR141 overexpressed cells upon rapamycin treatment (Figure 4G). This research unravels the enhanced expression of p53 in GPR141 overexpressed cells upon rapamycin treatment while restricting p-mTOR signaling. Overall, data from this study delineate that GPR141 aids in cancer cell proliferation through p53 degradation mediated by Cullin1 interaction and elevated p-mTOR1 levels, which showed a positive correlation with Cullin1.

### Silencing of GPR141 restricts cell proliferation and migration

**Figure 5:**
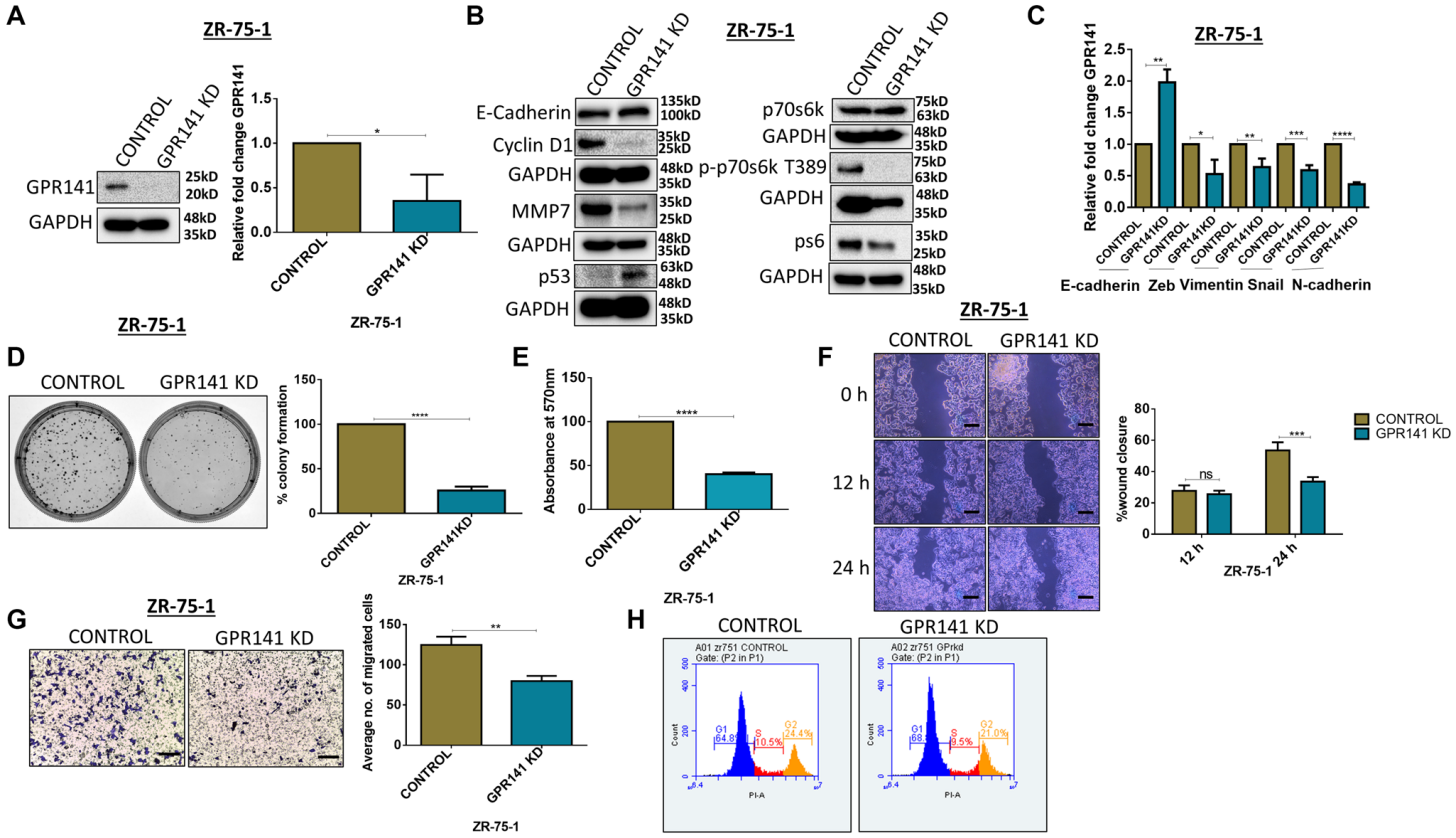
GPR141 silencing suppresses migration of breast cancer cells and tumorigenesis *in vitro*. (**A**) Immunoblot and relative mRNA expression showing the level of GPR141 in ctrl si-RNA and GPR141 si-RNA-treated ZR-75-1 cells. (**B**) The immunoblot shows the expression levels of oncogenic stimulators, p53, phosphorylated p-mTOR1 substrates and epithelial marker. (**C**) Relative mRNA expression of EMT markers after GPR141 silencing. (**D**) Clonogenic assay showing colony numbers per well after GPR141 silencing in ZR-75-1 cells. Graphical data were also represented. (**E**) Cell proliferation by 3-(4,5-dimethylthiazol-2-yl)-2,5-diphenyl-2H-tetrazolium bromide (MTT) assay at 48 h after GPR141 silencing. (**F**) Wound healing assay showing 0, 12, and 24 h post wounding and its graphical representation showing percentage wound closure in ctrl si-RNA and GPR141 si-RNA-treated ZR-75-1 cells. Scale bar: 50 μm. (**G**) Migration assay through transwell chamber 48 h post-stimulation with 5% FBS. Scale bar used in this study: 50 μm. (**H**) Fluorescence-activated cell sorting assay (FACS) shows an arrest in the G1 phase upon GPR141 silencing in ZR-75-1 cells. The data are represented as mean ± SD. Student’s *t*-test and two-way ANOVA were used for the statistical analysis, *n* = 3 (^****^ *P* ≤ 0.0001, ^***^ *P* ≤ 0.001, ^**^ *P* ≤ 0.01, ^*^ *P* ≤ 0.05, Significant). Abbreviation: ns: no significant difference.

Tumor cells employ several methods, such as altering their local environment and transforming their gene-expression patterns to generate angiogenic factors to meet their rising dietary and oxygen needs [28]. To determine the proliferative and migratory pattern in breast cancer cells, we performed siRNA-mediated knockdown of GPR141 in ZR-75-1 cells. Immunoblot and transcript data showed significant GPR141 suppression in ZR-75-1 cells (Figure 5A). Next, we assessed the role of silenced GPR141 in breast tumorigenesis by performing colony forming assay, transwell migration, and wound healing assay. Knockdown of GPR141 showed an increase in the expression of the epithelial marker, E-cadherin (Figure 5B, 5C), and enhanced p53 expression both at protein and transcript level (Figure 5B, Supplementary Figure 5A). GPR141 knockdown decreased the phosphorylation levels of p-mTOR1 substrates. Immunoblot also showed the total protein expression of m-TOR1 substrates (Figure 5B). Densitometry quantification has also been provided (Supplementary Figure 5B). Moreover, the knockdown of GPR141 deters cellular proliferation (Figure 5D, 5E) and migration (Figure 5F, 5G), G1 stage cell cycle arrest (Figure 5H), in breast cancer cells.

### GPR141 stimulates breast carcinoma progression and alters the tumor microenvironment *in vivo*

**Figure 6:**
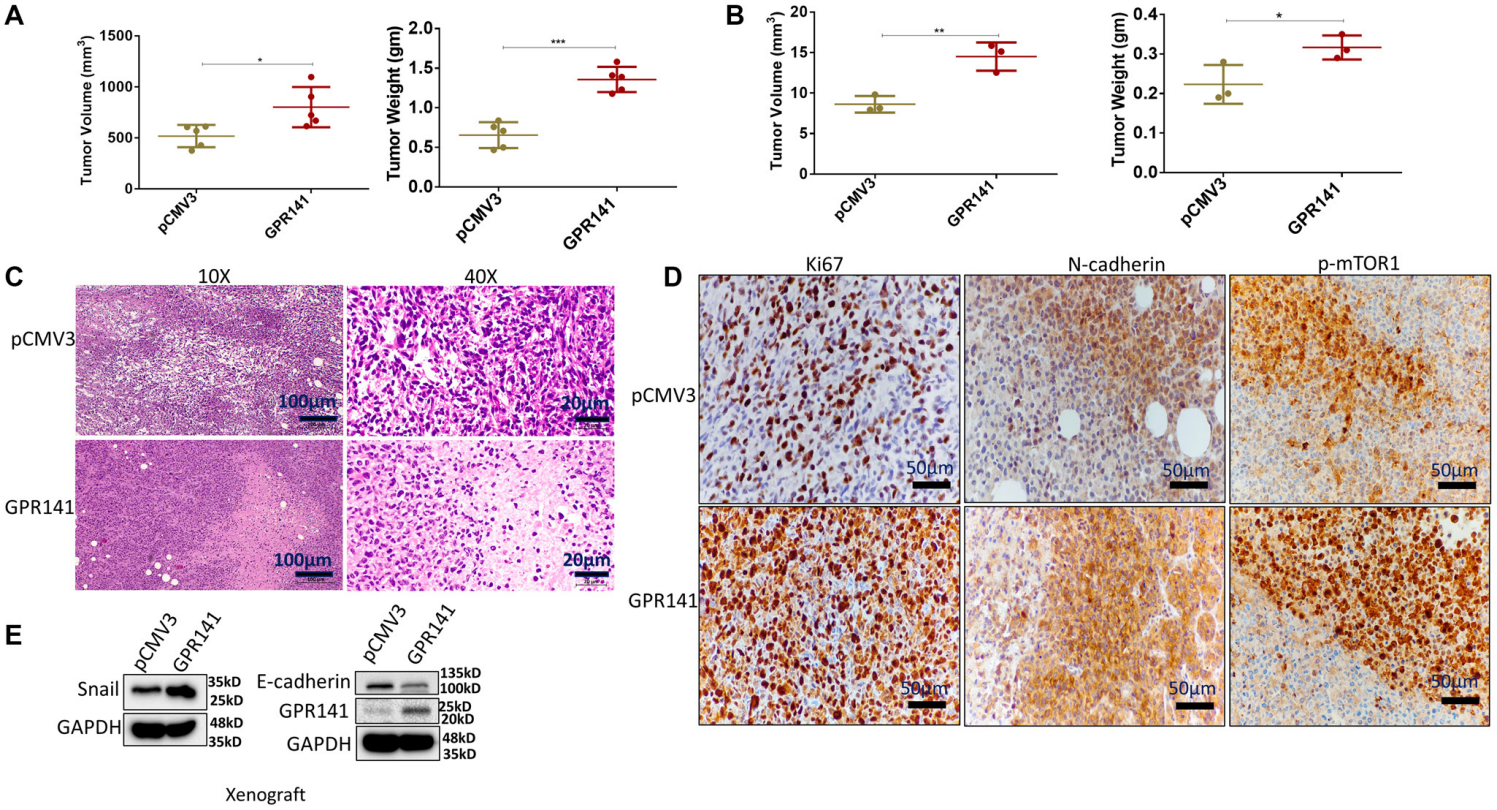
GPR141 overexpression in breast cancer cells increases their propensity to form tumors in female NOD SCID mouse and chick CAM models and modulates the tumor microenvironment *in vivo*. (**A**) Graphical illustration showing tumor volume and weight in female NOD SCID mice model. (**B**) Graphical data showing the tumor volume and weight in the chick CAM model. (**C**) Representative H&E images of tumor sections in NOD SCID female mice (10X, 40X) injected with control pCMV3 and overexpressed MDA-MB-231 cells orthotopically into the mammary fat pad of mice. The scale used for this study was 100 μm for 10X and 20 μm for 40X magnification. (**D**) Immunohistochemical analysis of Ki67 (proliferation marker), N-cadherin (mesenchymal marker), and p-mTOR1 in the mammary fat pad tumors. The scale used in the study- 50 μm for 20X image magnification. (**E**) Immunoblot showing expression of GPR141, epithelial and mesenchymal markers in xenograft tumors. The data are represented as mean ± SD. Student’s *t*-test was used for the statistical analysis, *n* = 3 for the chick CAM model, and *n* = 5 for SCID female mice model (^***^ *P* ≤ 0.001, ^**^ *P* ≤ 0.01, ^*^ *P* ≤ 0.05).

To investigate the function of GPR141 in tumor development *in vivo*, we administered control pCMV3 and GPR141 overexpressed MDA-MB-231 cells orthotopically into the mammary fat pad of female NOD SCID mice. The mice were euthanized after 70 days. Tumor volume and weight were measured. Additionally, the rat, mouse, and human GPR141 share more than 95% of the identical amino acids in the TM Regions, implying a very significant level of conservation [9]. Mice showed pronounced tumor growth in GPR141 overexpressed cells compared to the control cells (Figure 6A). Chick Chorioallantoic membrane (CAM) model is an established method to check cancer cell development, angiogenesis, and invasion [29]. We also showed tumor development in GPR141 overexpressed cells by the Chick CAM model (Figure 6B). H and E staining of the histological sections of the tumors were performed to confirm the arrangement and structure of the cells (Figure 6C). Next, Immunohistochemistry (IHC) analysis exhibited increased Ki67, N-cadherin, and p-mTOR1 levels in the tumors induced by GPR141 overexpressed cells compared to the tumors generated by control pCMV3 transfected cells (Figure 6D). The quantification of Ki67, N-cadherin, and p-mTOR1 positive cells has also been provided (Supplementary Figure 6). Western blot data showed increased Snail and decreased E-cadherin expression in GPR141 overexpressed xenograft (mouse) model (Figure 6E).

**Figure 7:**
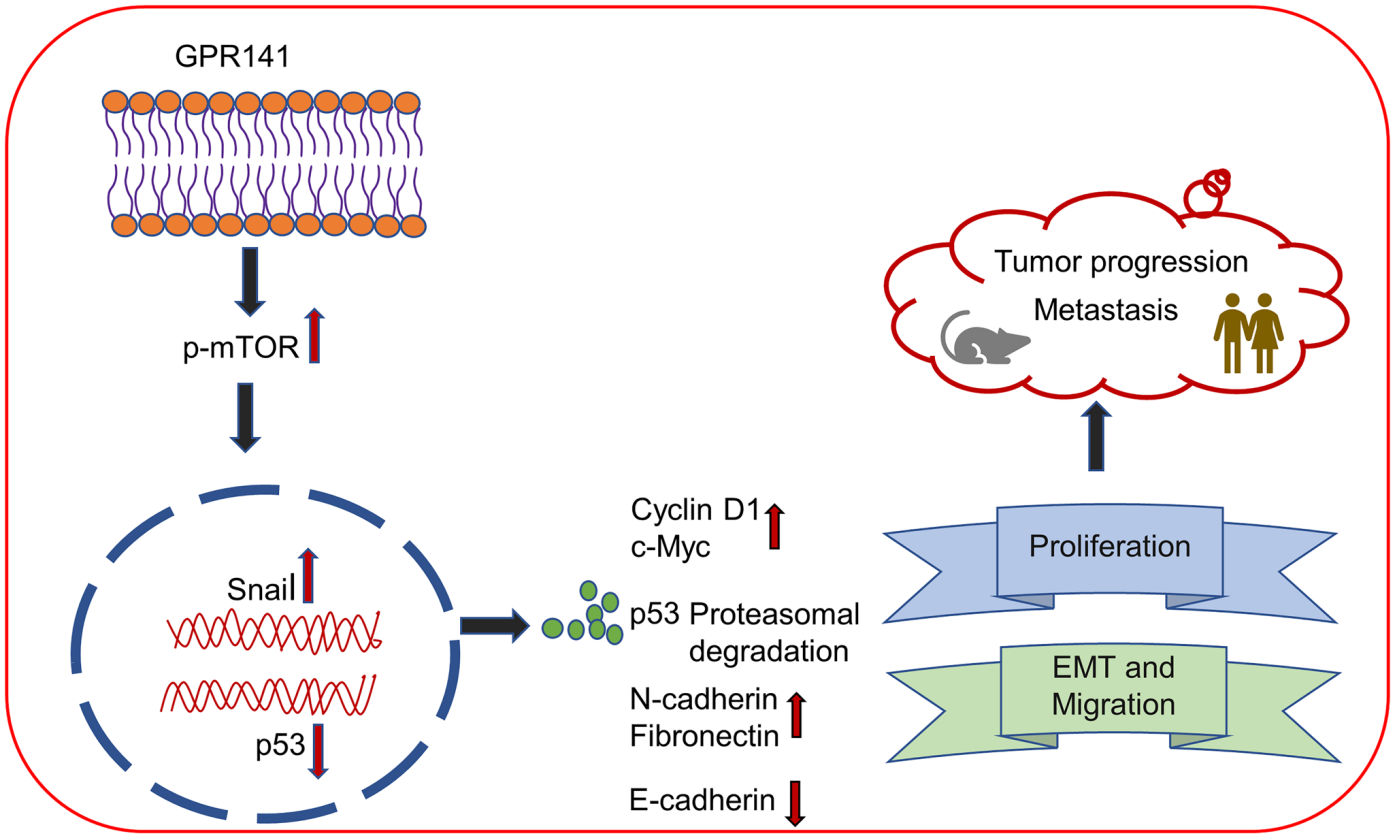
Schematic demonstrating the role of GPR141 in mediating breast cancer development and progression. (**A**) Schematic representation showing that GPR141 drives breast metastasis and tumorigenesis via modulating EMT markers, p-mTOR1/p53 signaling axis, oncogenic mediators’ expression, and tumor microenvironment.

In concordance with cancer progression, tumor sections also showed higher expression of proliferation marker, Ki67, and lower expression of E-cadherin on GPR141 transfected tumors compared to control tumors, confirming its involvement in GPR141-mediated breast cancer progression. Collectively our findings suggest that GPR141 regulates breast tumorigenesis via modulating p-mTOR1/p53 signaling, EMT biomarkers, oncogenes expression, and the tumor microenvironment *in vitro* and *in vivo*, as an annotated simulation model in detail (Figure 7).

## DISCUSSION

Aberrant GPCR signaling is shown to enhance the tumorigenic potential of cells by modulating migration, growth, and invasion [30]. Our findings demonstrate that GPR141 controls malignancy *in vitro* and *in vivo* in humans and xenografts (Chick and SCID mice). It is also evident that GPCRs’ erroneous expression or signal transduction in cancer cells can influence tumor malignancy [31]. Furthermore, we have shown enhanced tumor development in GPR141 overexpressed cells. Altogether, these research findings elucidated that GPR141 accelerates breast carcinogenesis by modulating EMT transition and tumor niche.

GPR141 governs cancer cells’ proliferative and migratory attributes via a plethora of signal transduction pathways, such as p-mTOR1 and p53 signaling. Our study shows an increased p53 expression upon MG132 treatment at the protein level, suggesting the proteasomal pathway partly mediates p53 degradation via Cullin1 upon GPR141 overexpression. GPR141 promotes the phosphorylation of mTOR1 (ser 2448) and its associated signaling pathways. Upon MG132 treatment, ps6 is downregulated, showing the sensitivity of mTOR1 signals to it. So, we tried to delineate the E3 ubiquitin ligase in charge of preserving the mTOR pathway. Since Cullin1 is a member of the E3 ubiquitin ligase family, we checked the interaction of Cullin1 with ps6. Co-IP data confirm the interaction of Cullin1 with ps6. By employing p-mTOR1 inhibitor rapamycin in breast cancer cells, we established the p-mTOR1 transduction pathways’ relevance in GPR141-mediated carcinogenesis. Moreover, rapamycin exhibits enhanced p53 expression compared to GPR141 overexpressed cells. Oxidative stress contributes to enhanced cellular ROS production by inhibiting p53 activity. p53 provides the antioxidant defense to maintain genome stability and inhibit ROS-activated signaling cascades such as mTOR leading to cancer cell proliferation [32]. Rapamycin inhibits ROS production in GPR141 overexpressed cells. The p-mTOR1/p53 axis is a crucial signaling mechanism implicated in GPR141-stimulated breast tumor growth and metastasis, according to our current studies, which established that GPR141 promotes breast carcinogenesis by p-mTOR1/p53 axis-dependent cellular proliferation and migration. At the post-translational and transcriptional levels, mTORC1 incorporates a variety of stimuli and transduction networks to accelerate protein, lipid, and nucleotide biosynthesis and block catabolic processes like autophagy [33]. GPR141 activation suppresses Beclin 1, thereby inhibiting autophagy by p-mTOR1.

One essential process in disseminating solid tumors is the epithelial-to-mesenchymal transition (EMT). Metastatic cascade is correlated with the induction of EMT transcriptional factors such as Snail and Twist1. Recently, various human malignancies exhibited a significant relation between EMT transcription factors and NF-κB stimulation [34]. Snail is a potent EMT modulator. It significantly restricts E-cadherin expression and upregulates EMT markers fibronectin, MMP7, and cyclin D1 in various tumors [35]. Our research findings established that GPR141 elevates the expression of potent mesenchymal markers (N-cadherin, Fibronectin, MMP7) together with oncogenic stimulators (c-Myc, NF-κB, and CyclinD1, D3) and transcription factor Snail. The tumor microenvironment is a central hub for various stromal cells, cancer cells, immune cells, and local secretory factors. The interplay between cancer cells and their milieu is vital for tumor growth, dissemination, and therapeutic intervention [36]. Angiogenesis, the mechanism of newly formed blood vessels originating from the pre-existing circulatory has been implicated in the development, progression, and metastasis of breast cancer [37]. GPR141 enhances the expression level of the angiogenic mediator Vascular endothelial growth factor (VEGF). Additionally, VEGF can aid in creating a vascular and immunosuppressive milieu [37]. Our results show that GPR141 facilitates migration and influences the tumor microenvironment. However, understanding the role of GPR141 in the crosstalk between tumor cells and their niche can be an intriguing treatment strategy to address multiple mechanism driving tumorigenesis.

In conclusion, our research highlights the gain of function of GPR141 drives breast tumorigenesis by inducing tumor cell properties via the p-mTOR1/p53 axis, altering EMT markers, and enhancing oncogenic mediators. GPR141 shows improved migratory behavior. GPR141 silencing impedes the migration of breast cancer cells. However, the role of GPR141 in the assessment of patient health outcomes in breast cancer is still lacking. Continued research into the biological functions and associated signaling events of the GPCRs underpinning tumor advancement and metastasis will aid in the establishment of new targets and innovative medicinal techniques for the treatment of patients with breast cancer [38]. TCGA database shows enhanced expression of GPR141 in leukemia, ovarian cancer, and head and neck cancer. GPR141 mutations and their significance in cancer need to be explored, which will provide a cutting-edge and successful therapeutic strategy beyond breast cancer too. Furthermore, GPR141 promotes breast tumor development *in vivo* and influences the tumor milieu to facilitate breast cancer progression. Our study indicates that GPR141 and its regulated signaling circuitry could be a promising therapeutic candidate for controlling breast cancer development and metastasis.

## MATERIALS AND METHODS

### Cell lines and cell culture

The human breast cancer cell lines MCF-7 (RRID:CVCL_0031), ZR-75-1 (RRID:CVCL_0588), T-47D (RRID:CVCL_0553), SKBR3 (RRID:CVCL_0033), MDA-MB-231 (RRID:CVCL_0062), and MDA-MB-468 (RRID:CVCL_0419) were procured from the National Repository of Animal Cell Culture, NCCS Pune (Maharashtra, India). MCF10A (RRID:CVCL_0598) cell line was obtained from The American Type Culture Collection (ATCC), USA. All cell lines were screened for *Mycoplasma* and tested negative for it. They were independently verified by short tandem repeat (STR) DNA fingerprinting at the Institute of Life Sciences (Bhubaneswar, India). Experiments were conducted within five passages post-thawing. MCF-7, ZR-75-1, SKBR3, and MDA-MB-231 cells were cultured in Dulbecco’s Modified Eagle’s Medium (DMEM), whereas T-47D, MDA-MB-468 in Roswell Park Memorial Institute 1640 (RPMI) were supplemented with 10% fetal bovine serum (FBS) and 100 U/ml penicillin-streptomycin (MP Biomedicals, Bengaluru, India). MCF-10A was cultured in DMEM F12 containing horse serum (5%), hydrocortisone (0.5 mg/ml), EGF (20 ng/ml), insulin (10 μg/ml), cholera toxin (100 ng/ml), and 100 U/ml penicillin-streptomycin. Cells were maintained at 37°C, 5% CO2, and 95% humidity.

### Plasmids and drugs

Human GPR141 cDNA clone expression plasmid was purchased from Sino Biologicals. MG132 and Chloroquine diphosphate were obtained from Tocris BioScience (1748, 4109). The stock solution was prepared in dimethyl sulfoxide (DMSO) for MG132 and water for chloroquine diphosphate. The final concentration of both the chemicals was kept at 2 μM in the culture medium. Rapamycin was purchased from Merck (R8781). Cells were treated with 20 nM rapamycin for 24 h to assess cell proliferation, viability, and migration assays.

### GPR141 promoter analysis, transfection, and luciferase assays

GPR141 promoter (1259 bp) was cloned into pGL3 luciferase vector (Addgene) by combining the forward primer with reverse primer, 5′-ACAGGTACCAGTTTCCTGATGCAGAGGC-3′ and 5′-ACACTCGAGTCACTGGTAACTTAGGGCTC-3′. The pGL3-GPR141, pEGFP-p53, p53 shRNA, and pRL-Renilla luciferase construct (Promega) was transfected into MDA-MB-231 cells by Xfect™ Transfection Reagent in different combinations according to the manufacture protocol. 48 h post-transfection, luciferase activity was quantified using a Dual luciferase assay detection kit (Promega), and the readings were normalized against the Renilla luciferase activity. Luciferase reading was measured in a luminometer (Sirius, Titertek-Berthold). The graph was plotted with normalized readings using GraphPad Prism software version 6.01.

### Clonogenic assay

An *in vitro* clonogenic assay was performed as earlier described [39] to analyze the single-cell growth potential of MCF-7, ZR-75-1, and MDA-MB-231 cells to assemble into a colony. GPR141 overexpressed, knockdown, and control MCF-7, MDA-MB-231, and ZR-75-1 cells were seeded at 5 × 10^2^ cells in 60 mm plates (Corning, Pune, India). The plates were incubated at 37°C, 5% CO_2,_ for 2 weeks to facilitate the growth of colonies (approximately 50 cells per colony) and then stained with 0.01% crystal violet. To determine colony formation rate, the stained cells in the form of colonies were dissolved in 10% (v/v) acetic acid, and the absorbance was quantified at 540 nm using Varioskan™ Flash Multimode Reader (Thermo Scientific). The values were calculated by the following formula- Colony formation rate = 100% × (experimental absorbance value/control absorbance value).

### Cell viability assay

Cell viability assay was conducted as previously described [40]. Breast cancer control, GPR141 overexpressed and knockdown cells were seeded in 96-well cell culture plates (Corning, Pune, India) at 3 × 10^3^ cells/well density. After 48 h incubation, 10 μL of MTT (MP Biomedical) (5 mg/mL in PBS) was added to each well and incubated at 37°C, 5% CO2 atmospheric condition for another 3 h. Then the medium was removed, and 100 μL of DMSO was added to dissolve the formed formazan crystals. The solubilized crystals were quantified by scanning the plates at 570 nm using Varioskan™ Flash Multimode Reader (Thermo Fisher Scientific, Bangalore, India).

### Transwell-migration assay

Transwell-migration assay was performed as earlier described [40] using Boyden’s chambers comprising polycarbonate filters with a pore size of 8 μm (BD Falcon, Bhubaneswar, India). MCF-7, ZR-75-1, and MDA-MB-231 cells were seeded at a density of 2.5 × 10^4^ cells in the upper chamber of a 12-well transwell system in 500 μL of serum and DMEM. The lower chamber supplemented with 5% serum was used as a chemo-attractant. After 24 h, the cells on both sides of the membrane were fixed with 10% formalin and stained with 0.01% crystal violet stain. The membrane was then washed with 1X PBS, and the cells attracted towards the serum were visualized under a Carl Zeiss inverted microscope (Carl Zeiss, Germany). The number of migrated cells in control, GP141 overexpressed, and knockdown in six different fields were calculated using ImageJ software, and the average value was represented in the graph.

### Wound healing assay

As previously described, wound healing assay was carried out [40]. 1 × 10^4^ MCF-7, MDA-MB-231, and ZR-75-1 cells were plated and grown to 90% confluence in 12-well plate (Falcon Becton Dickinson). In order to impede the proliferation and examine the migratory ability of breast cancer cells, the plated cells were serum starved for 48 h. Cells were then scratched with a sterile 200 μL pipette tip in each well. The cells were washed twice with 1X PBS, and the images were captured using an inverted microscope (Carl Zeiss, Germany) at different time points, initially starting with 0 h post rinsing with 1X PBS. The wound closure percentage was determined by comparing them to the control cells with the help of ImageJ software.

### Immunoblot analysis

The whole cell lysates from breast cancer cell lines (MCF 10A, MCF-7, ZR-75-1, T-47D, SKBR3, MDA-MB-231 and MDA-MB-468) were obtained by using RIPA buffer (500 mM NaCl, 5 mM MgCl2, 1% Na deoxycholate, 20 mM Tris-HCl (pH 8.0), 10% glycerol, 1 mM EDTA, 100 mM EGTA, 0.1% NP40, 1% Triton X-100, 0.1 M Na3VO4, 1X Protease inhibitor, 1X Phosphatase Inhibitor). Protein concentration was calculated by the Bradford protein assay method. Total protein extracts (40 μg) were electrophoresed in 10% SDS-polyacrylamide gels and transferred onto polyvinylidene difluoride (PVDF) membrane (GE Healthcare Life Sciences, Chalfont, UK). Blots were incubated with 5% skimmed milk (MP Biomedicals, India) for 1 h of blocking. Then the membrane was cut prior to incubation with primary antibodies and kept at 4°C overnight with gentle shaking (details of all antibodies and reagents are provided in the (Supplementary Table 1)). The membrane was then washed with 1X TBS-T and incubated with anti-rabbit or anti-mouse horseradish peroxidase-conjugated secondary antibody (1:5000 dilution) for 1 h. After washing, the blots were developed using Western Blot Chemiluminescence HRP Substrate (Takara Bio Inc.) in Chemidoc XRS+ molecular 228 imager (Bio-Rad, Hercules, CA, USA). The images were quantified using Image J software (NIH, Bethesda, MD, USA).

### Quantitative PCR

Total RNA was isolated from MCF-7, ZR-75-1, MDA MB-231, and GPR141 overexpressed and knockdown cells using Tri reagent (Sigma-Aldrich). A total of 500 ng was digested with DNase-I enzyme (Sigma-Aldrich) and was subjected to cDNA synthesis using PrimeScript™ 1st strand cDNA Synthesis Kit (Takara Bio Inc.,). Quantitative real time PCR was performed using primers provided in the (Supplementary Table 2). GAPDH was taken as an internal reference. The relative mRNA level or fold change for each gene compared to control was computed using the value of the cycle threshold (ΔΔCt values) [41]. The results were plotted using GraphPad Prism version 6.01.

### Gene silencing with small interfering RNAs (siRNAs)

For knockdown experiments, GPR141 siRNA human (sc-89687, Santa Cruz) transfection was done using Lipofectamine™ RNAiMAX Transfection Reagent according to the manufacturer’s instructions in appropriately treated ZR-75-1 breast cancer cells. Sequence of pooled siRNA duplexes used in the study is provided in the (Supplementary Table 2). All experiments involving GPR141si transfection were done within 72 h.

### Fluorescence-activated cell sorting (FACS) for cell cycle

Fluorescence-activated cell sorting experiment was performed as previously described [42]. MCF-7, ZR-75-1, and MDA-MB-231 cells were grown in 6 well plates (Corning, Pune, India) in DMEM supplemented with 10% heat-inactivated fetal bovine serum at 37°C for 24 h prior to transfection with pCMV3-GPR141 construct/GPR141 siRNA and were allowed to grow for 48 h. Cells were trypsinized, followed by washing with 1X PBS twice. Then the cells were fixed with 70% ethanol for 4 h. After fixation, cells were rinsed with ice-cold 1X PBS thrice and stained with propidium iodide (PI). Sorting was performed and analyzed using BD Accuri (BD Biosciences).

### Chromatin Immunoprecipitation (chIP)

Chromatin Immunoprecipitation was carried out as earlier described [42]. Breast cancer cells were fixed with 1% (v/v) formaldehyde followed by washing twice with 1X PBS. Then the cells were lysed in SDS lysis buffer (1% (w/v) SDS, 10 mM EDTA, 50 mM Tris-HCl (pH 8.1)) with protease inhibitor cocktail (Sigma-Aldrich) and were sonicated using Bioruptor ultrasonicator device (Diagenode S.A., Seraing, Belgium) at M2 amplitude strength. The sonicated samples were subjected to pre-clearing with protein A/G agarose beads (GE Healthcare Life Sciences). These pre-cleared samples were diluted with chIP dilution buffer (0.01% (w/v) SDS, 1.1% (v/v) Triton X-100, 1.2 mM EDTA, 16.7 mM Tris-HCl (pH 8.1), 167 mM NaCl) and divided into two equal parts IgG and IP. 50 μl was taken as input and was stored at −80°C. The IgG and IP were incubated with 1μg of anti-IgG (Diagenode) and anti-p53 (GTX128135, GeneTex) antibodies, respectively. The protein-antibody complex was extracted by incubating the samples with protein A/G agarose beads. The protein-antibody-bead complex was extracted, and washed with a series of different washing buffers i.e. Low salt buffer (0.1% (v/v) SDS, 2 mM EDTA, 1% (v/v) Triton X-100, 20 mM Tris-HCl (pH 8.1) and 150 mM NaCl), High salt buffer (0.1% (v/v) SDS, 1% (v/v) Triton X-100, 2 mM EDTA, 20 mM Tris-HCl (pH 8.1) and 500 mM NaCl), LiCl salt buffer (0.25 M LiCl, 1% (v/v) NP-40, 1% (w/v) deoxycholic acid (sodium salt), 1 mM EDTA and 10 mM Tris-HCl (pH 8.1)), 1X TE (10 mM Tris-HCl (pH 8.1) and 1 mM EDTA) and were eluted using elution buffer (1% (v/v) SDS, 0.1 M NaHCO_3_). The eluted samples and input were reverse crosslinked with 5 M NaCl for 6 h at 65°C, followed by incubation with 0.5 M EDTA, 1 M Tris-HCl (pH 6.5), and proteinase K at 45°C for 1 h. chIP elutes were purified using phenol/chloroform, and ethanol precipitated. DNA samples were further used to perform PCR analysis to confirm the binding of p53 on the GPR141 promoter. The primer sequences used for chIP-PCR are GPR141 chIP Forward primer: 5′-ATGGCAGAAAGAACGCACATCTGCTG-3′ and Reverse primer: 5′-CAAAATGATACTGGCCCTGGGTTCT-3′.

### Co-immunoprecipitation

Co-immunoprecipitation was performed as previously described with minor modifications [43]. Cells were washed with 1X PBS (pH 7.4) twice and lysed with NP40 buffer (50 mM Tris-Cl pH 8.0, 150 mM NaCl, 1% NP40). Lysates were precleared by the addition of 50 μl of A/G agarose beads (GE Healthcare Life Sciences) for 2 h. Total protein (600 μg) and 5 μg of antibody were used for each IP and rotated overnight at 4°C. Beads (30 μg) were added to each IP and rotated for 2 h, followed by centrifugation at 500 × g for 3 min. Supernatants were removed, and pellets were washed four times with NP40 buffer. Complexes were eluted in SDS lysis buffer.

### Reactive oxygen species (ROS) detection assay

According to the manufacturer’s protocol, ROS level was assessed with CheKine™ Reactive Oxygen Species (ROS) Detection Fluorometric Assay Kit (Abbkine). 2′,7′-Dichlorofluorescein diacetate (DCFH-DA) 2 × 10^6^ control pCMV3 and GPR141 overexpressed MCF-7 and MDA-MB-231 cells, with or without rapamycin were washed with serum-free medium twice. 10 μM DCFH-DA was added onto 60 mm plates. The cells are allowed to incubate for 2 h at 37°C in the dark. After incubation, the cells were rinsed twice with a serum-free medium. The cells were trypsinized and resuspended with 1X DPBS. The fluorescence intensity was measured through flow cytometry in BD LSR Fortessa SORP (BD Biosciences).

### Experimental animal models

NOD SCID female mice and the Chick CAM model were used for the xenograft study. The NOD SCID mice were procured from Tata Memorial Centre (Advanced Centre for Treatment, Research, and Education in Cancer, Navi Mumbai, India). Animal Licence number for NOD SCID Mice: ILS/IAEC-215-AH/APR-21. Animals were maintained in compatible groups in individually ventilated cages (Citizen Industries, Ahmedabad, India) with corncob bedding, and wood shredding nesting material and fed with a standard laboratory rodent diet (VRK Nutritional Solution, India) and water *ad libitum*. The animal room was provided with a heating, ventilation, and air-conditioning (HVAC) system. The temperature was maintained at 22 ± 2°C and relative humidity at 50–70%. The room air changes per hour were 16–20. The mice were maintained at 12 h light:12 h dark light cycle in a noise-free environment. The mice were handled and used for experiments in accordance with the Committee for the Purpose of Control and Supervision of Experiments on Animals (CPCSEA) guidelines after obtaining ethical approval from the Institutional Animal Ethics Committee of the Institute of Life Sciences, Bhubaneswar. Fertilized eggs of seven days old were purchased from Central Poultry Development Organization, Bhubaneswar, India. Fertilized eggs were maintained in a 37°C Incubator. The experiment was conducted within 12 days in accordance with the Committee for the Purpose of Control and Supervision of Experiments on Animals (CPCSEA) guidelines.

### *In vivo* tumorigenesis

Four- to six-weeks old NOD SCID female mice (body weight, 22–25 g) were used for the xenograft study. The experimental protocols were approved by the Institutional Animal Ethical Committee (Institute of Life Sciences, Bhubaneswar, India), and all experiments were performed following the approved guidelines and regulations. Control and GPR141 overexpressed MDA-MB-231 cells (1 × 10^6^ cells) in Matrigel were injected orthotopically into the mammary fat pad of the mice. The experiment was terminated after 10 weeks, and the mice were euthanized. The Chick CAM model (7 days fertilized eggs) was also used for the tumor study *in vivo*. The experiment was concluded 5 days after tumor growth. Tumor volume was measured once a week with a digital caliper. The formula for calculation was tumor volume = 1/2(length × width^2^). The tumor specimens were fixed in 10% formalin, embedded in paraffin, and sectioned at 5 μm for histopathological studies.

### Histopathology and Immunohistochemistry

Histopathology and Immunohistochemistry experiments were conducted as previously described [39]. Tumor tissue samples were fixed in 10% formaldehyde solution before being embedded in paraffin wax. Paraffin-embedded tissue was sectioned into 5 μm slices and mounted on positively charged slides. Then the sections were deparaffinized in xylene and hydrated (100% EtOH, 95% EtOH, 70% EtOH, 2× H2O (2 min each), 3× distilled H2O (2 min each)). The sections were stained with Harris’ hematoxylin solution and washed with distilled water. Further, those sections were counter-stained with eosin, followed by dehydration, xylene, and DPX mounting. For the immunohistochemical assay, 3% hydrogen peroxide in methanol was used for endogenous peroxidase blocking. These sections were probed with N-cadherin (SC-7870), Ki67 (9449S), and p-mTOR1 (GTX132803) primary antibody overnight at 4°C. Tumor tissue sections slides were subsequently incubated with HRP-tagged secondary antibody. Staining was visualized with Diaminobenzidine (DAB) and counter-stained with hematoxylin. The images were taken using a Leica microscope (Leica DM500).

### Public domain data

Differential mRNA GPR141 gene expression was obtained from TNM plot via TCGA dataset (https://tnmplot.com/analysis/). cBioPortal illustrates disease-specific survival of breast cancer patients with GPR141 alteration and also analyzes the correlation of mTOR and TP53 with GPR141 (https://www.cbioportal.org/). The correlation of CUL1 expression with GPR141 expression was investigated through the StarBase v3.0 project (https://starbase.sysu.edu.cn/panCancer.php).

### Statistical analysis and reproducibility

Two-tailed paired Student’s *t*-test and two-way ANOVA were conducted to test the statistical significance of differences between the experimental groups. Differences in data with values of ^*^ *P* ≤ 0.05, ^**^ *P* ≤ 0.005, ^***^ *P* ≤ 0.001, and ^****^ *P* ≤ 0.0001 were considered statistically significant. Data are presented as mean ± standard deviation (SD) and analyzed using Microsoft Excel and Graph pad Prism 6.01 (GraphPad Software, La Jolla, CA, USA).

## SUPPLEMENTARY MATERIALS


