# Supplementary material for: G-protein-coupled receptor 141 mediates breast cancer proliferation and metastasis by regulating oncogenic mediators and the p-mTOR/p53 axis
Source: Oncotarget. 2023 May 19;14:466–80. doi: 10.18632/oncotarget.28433 (PMC10197958; doi:10.18632/oncotarget.28433)
Supplement: Supplementary file 1 [file oncotarget-14-28433-s001.pdf]

G-protein-coupled receptor 141 mediates breast cancer proliferation and metastasis by regulating oncogenic mediators and the p-mTOR/p53 axis

SUPPLEMENTARY MATERIALS

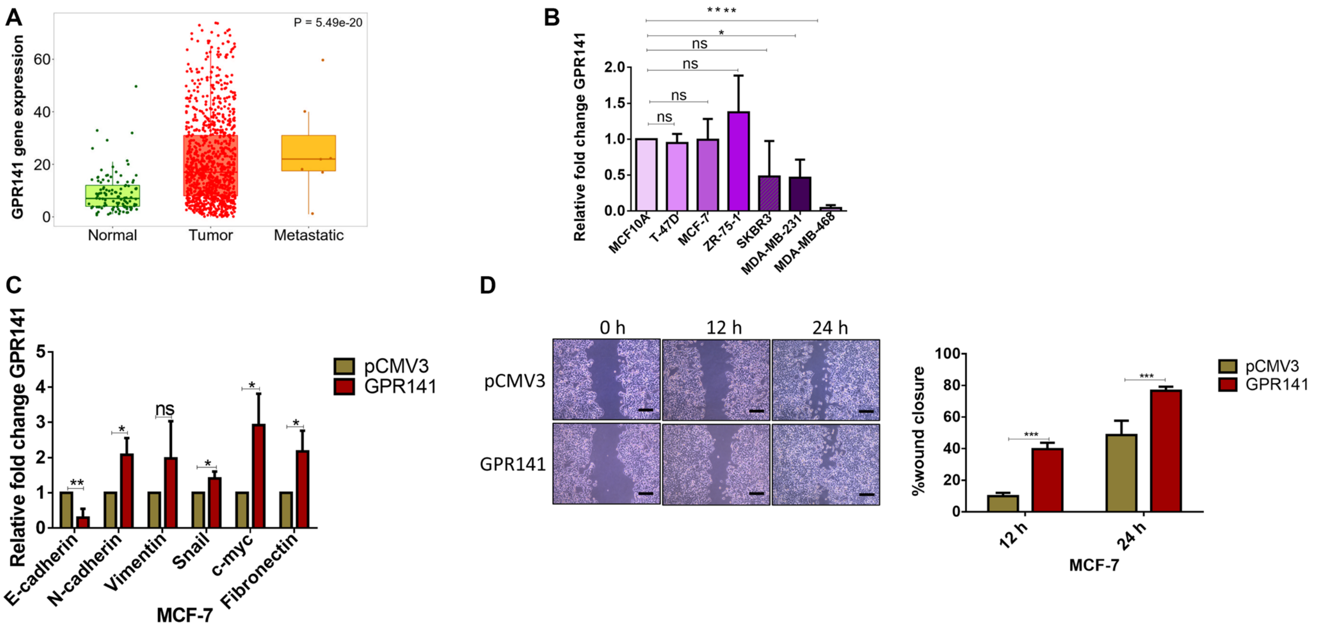

Supplementary Figure 1: Pronounced G-protein-coupled receptor 141 (GPR141) expression in breast cancer thereby facilitating migratory phenotype in breast cancer cells. (**A**) Box plot represents a comparison of GPR141 gene expression among normal, tumor, and metastatic breast samples through TNM plot analysis. Statistical significance was computed using Kruskal–Wallis tests. (**B**) qRT-PCR data shows expression level of GPR141 in different breast cancer cells and normal breast epithelial MCF10A cells. (**C**) Relative mRNA level of various oncogenes and EMT markers in ctrl pCMV3 and overexpressed GPR141 MCF-7 cells. (**D**) Wound healing assay of 0, 12, and 24 h post scratch showing percentage wound closure in ctrl pCMV3 and GPR141 overexpressed MCF-7 cells. Scale bar: 50 µm. Data are represented as mean ± SD. Student's *t*-test and two-way ANOVA were used for the statistical analysis, $n = 3$ ($^{****}P \leq 0.0001$, $^{***}P \leq 0.001$, $^{**}P \leq 0.01$, $^{*}P \leq 0.05$, Significant). Abbreviation: ns: no significant difference.

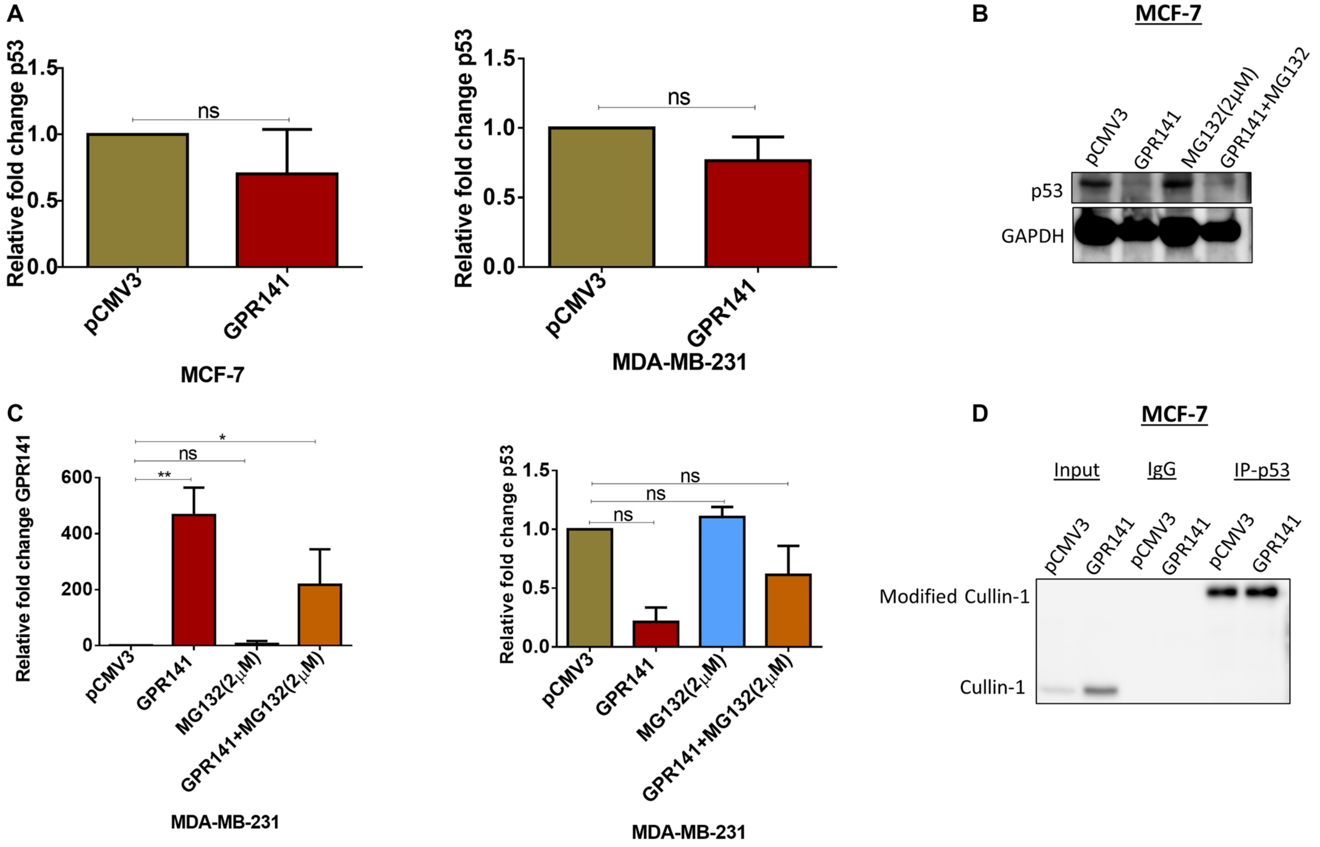

Supplementary Figure 2: GPR141 stimulates breast cancer cell proliferation by modulating p53 expression. (**A**) Relative p53 mRNA expression level MCF-7 and MDA-MB-231 cells control and GPR141 overexpressed were analysed using RT-qPCR. Three technical repeats ($n = 3$) were performed and the data represent the means ± SD. (two-tailed t test; ns: no significant difference (**B**) MCF-7 cells were treated with 2 µM MG132 and Chloroquine independently for 12 h and the p53 protein expression was analyzed by western blotting. Representative Western blot is shown. GAPDH was used as a loading control. ($n = 3$). (**C**) Relative mRNA expression of GPR141 and p53 in MG132 treated control and GPR141 overexpressed MDA-MB-231 cells. (**D**) Using immunoblotting, coimmunoprecipitation was performed with an antibody against p53 and analyzed with Cullin 1 antibody in both control pCMV3 and GPR141 overexpressed MCF-7 cells. An anti-IgG antibody was used as a negative control. All the data are represented as mean ± SD. Student's t-test was used for the statistical analysis, $n = 3$ ($^{**}P \leq 0.01$, $^{*}P \leq 0.05$, Significant). Abbreviation: ns: no significant difference.

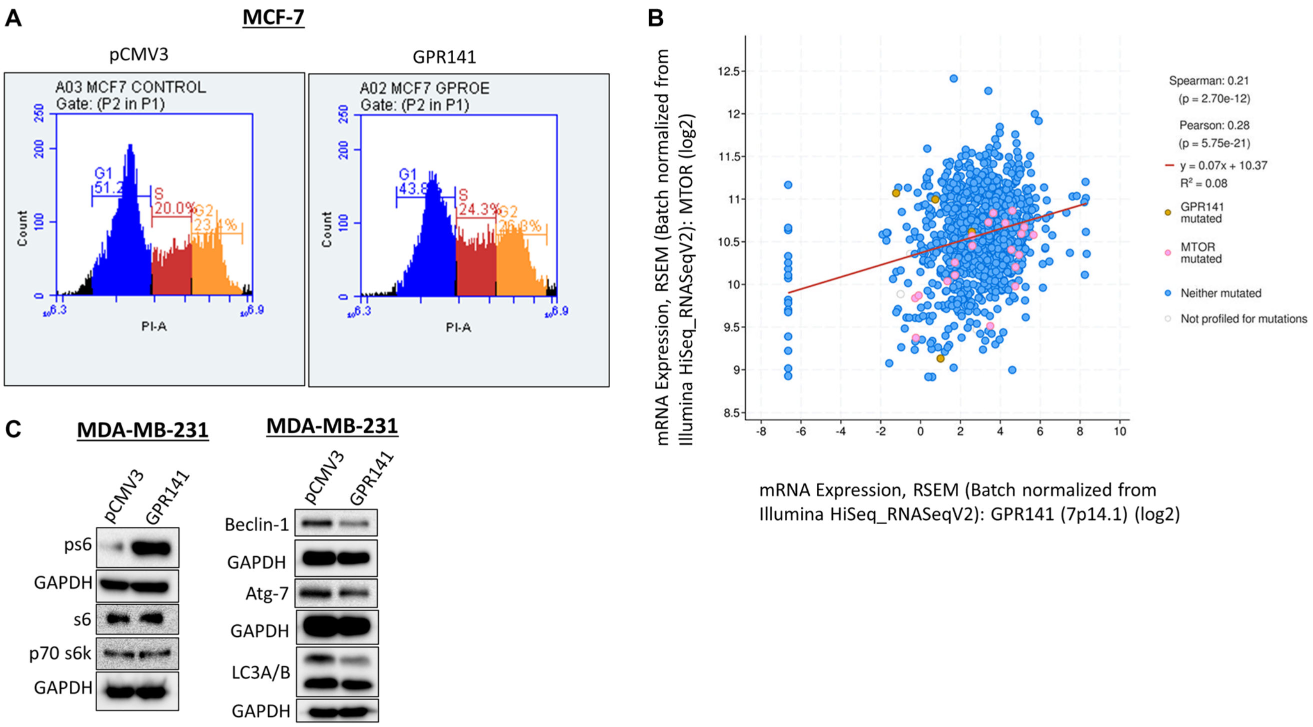

Supplementary Figure 3: GPR141 promotes cell cycle progression in breast cancer cells. (**A**) Fluorescence-activated cell sorting assay (FACS) showing an increase in G1-S transition phase upon overexpression of GPR141 in MCF-7 cells. (**B**) The correlation of mTOR mRNA expressions with GPR141 mRNA expressions in 1084 TCGA breast invasive carcinomas from cBioPortal. (**C**) Immunoblot showing the phosphorylation levels of p-mTOR1 and its substrates ps6, p70 s6kinase followed by autophagy markers expression in MDA-MB-231 cells after GPR141 overexpression.

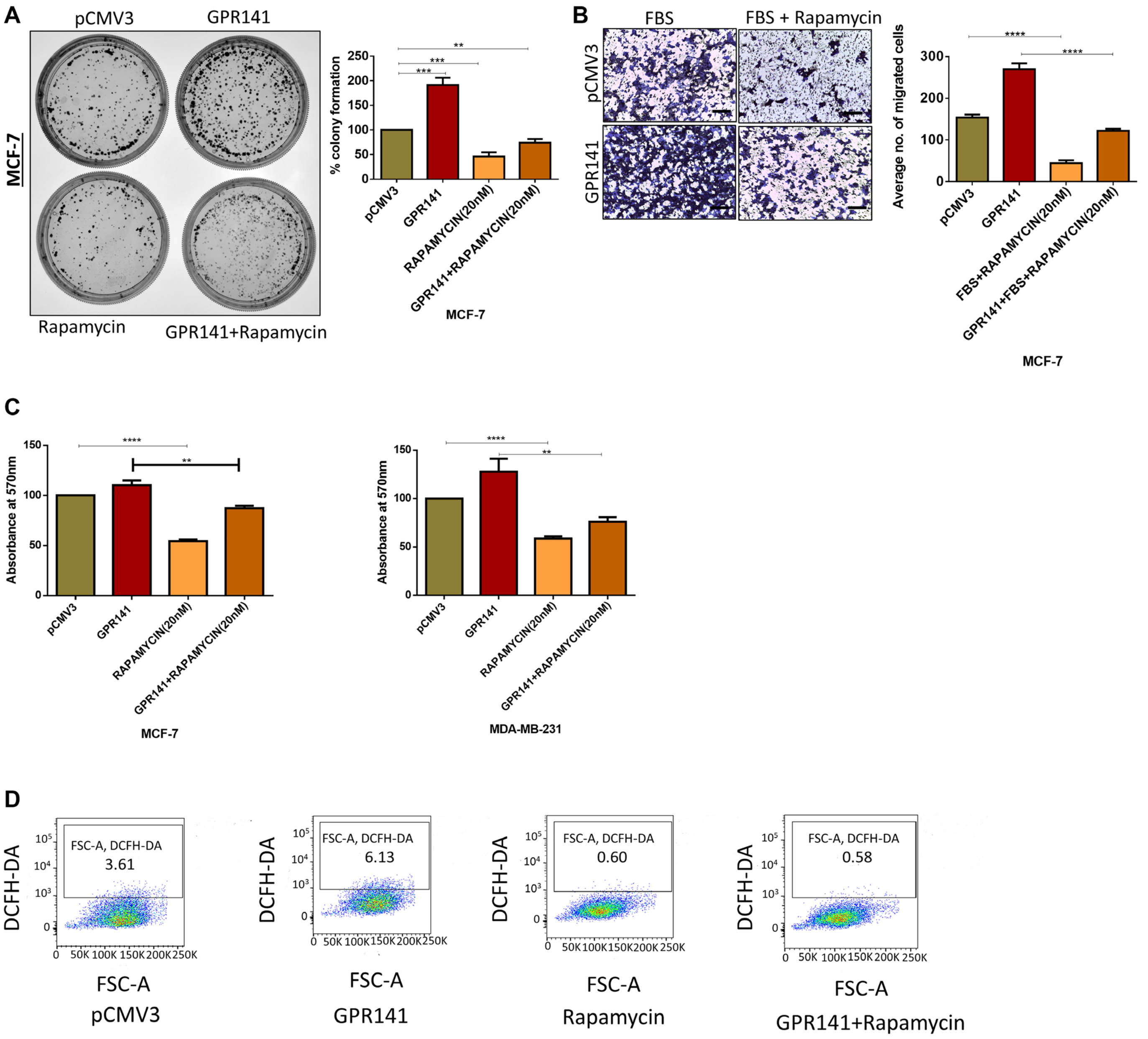

Supplementary Figure 4: GPR141 induces breast tumorigenesis by regulating the phosphorylated form of the mammalian target of rapamycin complex1 (p-mTOR1) signaling. (**A**) Colony forming assay showing the number of colonies developed with or without pmTOR1 inhibitor, rapamycin in MCF-7 cells. (**B**). Migration assay showing the number of migrated cells through transwell chamber 48 h after stimulation with 5% FBS in ctrl pCMV3 and GPR141 overexpressed MCF-7 cells with and without rapamycin. Scale bar: 50 µm. (**C**) Cell proliferation at 48 h with or without rapamycin treatment. (**D**) Reactive Oxygen Species (ROS) level in MCF-7 cells measured through 2′,7′-Dichlorofluorescein diacetate (DCFH-DA) at 24 h after rapamycin treatment. Data are represented as mean ± SD. Student's *t*-test was used for the statistical analysis, $n = 3$ ($^{****}P \leq 0.0001$, $^{***}P \leq 0.001$, $^{**}P \leq 0.01$, Significant).

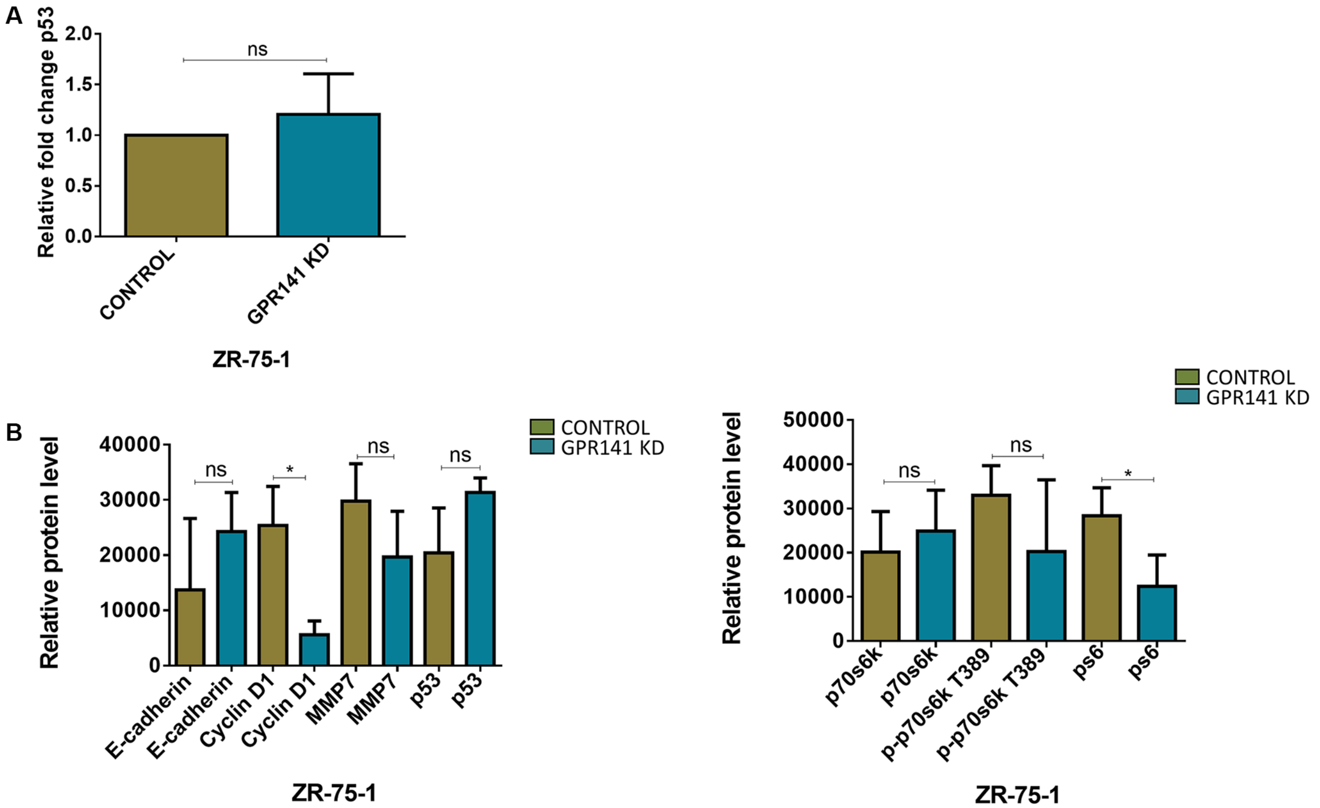

Supplementary Figure 5: GPR141 silencing restricts proliferation and migration in breast cancer cells. (**A**) Graphical data showing increased p53 expression at the transcript level upon GPR141 knockdown in ZR-75-1 cells. (**B**) Densitometry quantification of E-cadherin, Cyclin D1, MMP7, p53, p70s6k, p-p70s6k and ps6 has been provided for control and GPR141 knockdown ZR-75-1 cells. Data are represented as mean ± SD. Student's *t*-test was used for the statistical analysis, $n = 3$ ($^{*}P \leq 0.05$, Significant). Abbreviation: ns: no significant difference.

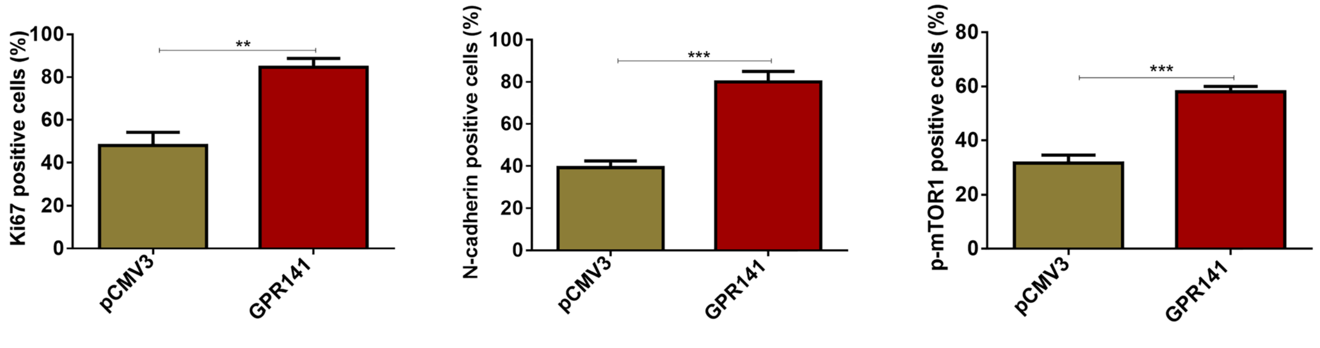

Supplementary Figure 6: GPR141 stimulates breast tumorigenesis *in vivo*. Graph demonstrates percentage of Ki67, N-cadherin and p-mTOR1 positive cells in control and GPR141 overexpressed tumor model. Scoring has been performed by a pathologist. Data are represented as mean ± SD. Student's *t*-test was used for the statistical analysis, $n = 3$ ($^{***}P \leq 0.001$, $^{**}P \leq 0.01$, Significant).

Supplementary Table 1: List of antibodies and reagents

Antibodies	Anti-GPR141	MyBioSource	MBS9604311
	Anti-N cadherin	Abcam	EPR1791-4
	Anti-E cadherin	Abcam	EP700Y
	Anti-p53	GeneTex	GTX102965
	Anti-mTOR(phosphoSer2448)	GeneTex	GTX132803
	Anti-Fibronectin	SantaCruz	sc-59826
	Anti-NFκB p65	SantaCruz	sc-514451
	Anti-GAPDH	SantaCruz	sc-32233
	Anti-Ki67	Cell signaling	9449S
	Anti-c-myc(D84C12)	Cell signaling	5605S
	Anti-pAKT1 (D7F10)	Cell signaling	9018
	Anti-AKT1 (2H10)	Cell signaling	2967
	Anti-ps6(Ser235/236)	Cell signaling	2211S
	Anti-s6	Cell signaling	2217T
	Anti-p70 s6 kinase	Cell signaling	9202S
	Anti-p-p70 s6 kinase (Thr389)	Cell signaling	9234T
	Anti-mTOR (7C10)	Cell signaling	2983T
	Anti-mTOR(phosphoSer2448)	Cell signaling	5536T
	Anti-Cyclin D1	Cell signaling	2978T
	Anti-Cyclin D3	Cell signaling	2936T
	Anti-p21 Waf/Cip1	Cell signaling	2947T
	Anti-p27 Kip1	Cell signaling	3686T
	Anti-p18 INK4C	Cell signaling	2896T
	Anti-CDK4	Cell signaling	12790T
	Anti-p44/42 MAPK	Cell signaling	9102S
	Anti-Beclin-1	Cell signaling	3495T
	Anti-Atg7	Cell signaling	8558T
	Anti-LC3A/B	Cell signaling	12741T
	Anti-Snail	Cell signaling	3430
	Anti-alpha tubulin (DM1A)	Novus	NB100-690
	Anti-PTEN Recombinant	Abcam	Y184
	Anti-MMP-7	SantaCruz	sc-515703
	Anti-Cullin 1	Cloud-Clone	PAC412Hu01
	Anti-HIF1a	Cloud-Clone	MAA798Hu22
	Anti-rabbit HRP conjugated secondary antibody	Cell signaling	7074P2
	Anti-mouse HRP conjugated secondary antibody	R&D	HAF007

Reagents	4, 5-dimethylthiazol-2yl)-2, 5-diphenyl tetrazolium bromide (MTT)	MP Biomedical	194592
	Propidium Iodide	MP Biomedical	195458
	Protease Inhibitor cocktail	Sigma	P2714-1BTL
	Trizol	Sigma	T9424
	Bradford Reagent	Sigma	B6916
	Skimmed Milk	Himedia	RM1254-500G
	Matrigel	Corning	354248
	Lipofectamine RNAiMAX	Thermo Fisher	13778150
	Xfect™ Transfection Reagent	Takara	631318
	Western BLoT Chemiluminescence HRP Substrate	Takara	T7101A
Kits and others	PrimeScript™ 1st strand cDNA Synthesis Kit	Takara	6110A
	TB Green® Premix Ex Taq™ II (Tli RNase H Plus)	Takara	RR82WR
	QIAprep Spin Miniprep Kit (250)	QIAGEN	27106
	Dnase I Kit	Sigma	AMPD1-1KT
	Boyden's chambers (0.8 μm)	BD Falcon	353182
	PVDF membrane	Merck	IPVH00010
	Dual Luciferase Reporter Assay	Promega	E1910
Mouse model	NOD.CB17-Prkdcsid/NCrCrl- Female	Charles River	

Supplementary Table 2: List of primers and GPR141 siRNA duplexes

Primer name	Oligo sequences (5′–3′)
GPR141 PROMOTER FW KpnI	ACAGGTACCAGTTTCCTGATGCAGAGGC
GPR141 PROMOTER RV XhoI	ACACTCGAGTCACTGGTAACTTAGGGCTC
GPR141 chIP FW	ATGGCAGAAAGAACGCACATCTGCTG
GPR141 chIP RV	CAAAATGATACTGGCCCTGGGTTCT
GPR141 RT FW	GATCCTGGTCACCAGATACCTC
GPR RT RV	AATCACCAGCGTCCACATGCCA
Cyclin D1 RT FW	GCTCCTGTGCTGCGAAGTGGAA
Cyclin D1 RT RV	TTTGAAGTAGGACACCGAGGGCG
p53 RT FW	ACCCAGGTCCAGATGAAG
p53 RT RV	CACTCGGATAAGATGCTGA
GAPDH RT FW	ATGGGGAAGGTGAAGGTCG
GAPDH RT RV	GGGGTCATTGATGGCAACAATA
E-cadherin RT FW	GACAACAAGCCCGAATT
E-cadherin RT RV	GGAAACTCTCTCGGTCCA
Fibronectin RT FW	CAGTGGGAGACCTCGAGAAG
Fibronectin RT RV	TCCCTCGGAACATCAGAAAC
N-cadherin RT FW	CGGGTAATCCTCCCAAATCA
N-cadherin RT RV	CTTTATCCCGGCGTTTCATC

Snail RT FW	GCAAATACTGCAACAAGG
Snail RT RV	GCACTGGTACTTCTTGACA
ZEB1 RT FW	TGCACTGAGTGTGGAAAAGC
ZEB1 RT RV	TGGTGATGCTGAAAGAGACG
Vimentin RT FW	GAGAACTTTGCCGTTGAAGC
Vimentin RT RV	GCTTCCTGTAGGTGGCAATC
c-Myc RT FW	CTTCTCTCC GTCCTCGGATTCT
c-Myc RT RV	GAAGGTGATCC AGACTCTGACCTT
**GPR141 siRNA duplex name with catalog number**	**Oligo sequences (5′–3′)**
GPR141 siRNA sc-89687A Sense	CCAUGGCGGUCAUUAACUUTT
GPR141 siRNA sc-89687A Anti-Sense	AAGUUAAUGACCGCCAUGGTT
GPR141 siRNA sc-89687B Sense	GGAAUCCAUGAGGAAUACATT
GPR141 siRNA sc-89687B Anti-Sense	UGUAUUCCUCAUGGAUUCCTT
GPR141 siRNA sc-89687C Sense	CUACGCCACUCUUUACUAUTT
GPR141 siRNA sc-89687C Anti-Sense	AUAGUAAAGAGUGGCGUAGTT
